# Artery-vein specification in the zebrafish trunk is pre-patterned by heterogeneous Notch activity and balanced by flow-mediated fine-tuning

**DOI:** 10.1242/dev.181024

**Published:** 2019-08-27

**Authors:** Ilse Geudens, Baptiste Coxam, Silvanus Alt, Véronique Gebala, Anne-Clémence Vion, Katja Meier, Andre Rosa, Holger Gerhardt

**Affiliations:** 1 Vascular Patterning Laboratory, Center for Cancer Biology, VIB, Leuven B-3000, Belgium; 2 Vascular Patterning Laboratory, Center for Cancer Biology, Department of Oncology, KU Leuven, Leuven B-3000, Belgium; 3 Integrative Vascular Biology Laboratory, Max-Delbrück Center for Molecular Medicine in the Helmholtz Association (MDC), Robert-Rössle-Strasse 10, Berlin 13125, Germany; 4 DZHK (German Center for Cardiovascular Research), partner site Berlin; 5 Berlin Institute of Health (BIH), Berlin, Germany

**Keywords:** Notch, Angiogenesis, Artery, Developmental patterning, Haemodynamic, Vein

## Abstract

How developing vascular networks acquire the right balance of arteries, veins and lymphatic vessels to efficiently supply and drain tissues is poorly understood. In zebrafish embryos, the robust and regular 50:50 global balance of intersegmental veins and arteries that form along the trunk prompts the intriguing question of how does the organism keep ‘count’? Previous studies have suggested that the ultimate fate of an intersegmental vessel (ISV) is determined by the identity of the approaching secondary sprout emerging from the posterior cardinal vein. Here, we show that the formation of a balanced trunk vasculature involves an early heterogeneity in endothelial cell behaviour and Notch signalling activity in the seemingly identical primary ISVs that is independent of secondary sprouting and flow. We show that Notch signalling mediates the local patterning of ISVs, and an adaptive flow-mediated mechanism subsequently fine-tunes the global balance of arteries and veins along the trunk. We propose that this dual mechanism provides the adaptability required to establish a balanced network of arteries, veins and lymphatic vessels.

## Abstract (precis)

**Highlighted Article:** A stepwise dual mechanism involving Notch signalling and flow provides the adaptability required to establish a balanced network of arteries and veins in the zebrafish trunk.

## INTRODUCTION

Efficient supply of oxygen and nutrient to tissues and organs is dependent on the formation of a hierarchically branched blood vessel network, comprising feeding arteries, capillaries and draining veins. During zebrafish development, the first axial artery and vein assemble from progenitor cells guided by local cues in the tissue (Kohli et al., 2013). However, the subsequent expansion of vascular networks sees arteries and veins arise through the sprouting and remodelling from the primitive vascular plexus (Isogai et al., 2003). When and where to form an artery or vein is a complex biological problem, as endothelial cells (ECs) adopt distinct gene expression repertoires associated with specific morphogenic behaviours in arteries and veins (Torres-Vazquez, Kamei and Weinstein, 2003). Organ-specific signatures additionally contribute to EC heterogeneity. The complexity of these differentiation processes and the multitude of chemical and physical morphogenic cues applied to the network appear to provide a daunting task for patterning (Nolan et al., 2013). Observations of stereotyped branching patterns of arteries and veins suggested that localised guidance cues drive artery formation (Carmeliet and Tessier-Lavigne, 2005). At the same time, blood flow is a crucial determinant of artery-vein formation in the chick and mouse yolk sac, and appears to be essential for all aspects of vascular remodelling and plasticity (le Noble et al., 2004; Lucitti et al., 2007). Rerouting flow is able to shape new arteries, and the plasticity reported after infarct illustrates that even rerouting flow in the established network triggers plastic responses (le Noble et al., 2004). The fact that many genes are differentially regulated by shear stress in ECs following exposure to blood flow suggests that genetic regulation and flow-dependent mechanisms are not necessarily exclusive, but integrated (Lehoux and Tedgui, 2003; Wragg et al., 2014). Yet how this is achieved and coordinates the correct number, branching pattern and spacing of arteries and veins remains largely unknown.

In the zebrafish trunk vasculature, the question takes on an additional dimension, as it first arises as an all-arterial network and is subsequently remodelled into a balanced network of arteries and veins. Although the order of arteries and veins along the trunk is not fixed, every embryo forms a balanced number of arteries and veins (Bussmann et al., 2010). How exactly this remodelling is organised to result in this balance is currently unknown. The intersegmental vessels (ISVs) initially arise as arterial vessels but then remodel into veins, or alternatively remain arterial and guide lymphatic structures (Geudens et al., 2010; Isogai et al., 2003; Yaniv et al., 2006). This process has been described to follow local cues in the tissue, and gene regulatory mechanisms are thought to drive fate decisions into artery, vein or lymphatic structures. The main axial vessels, the dorsal aorta (DA) and posterior cardinal vein (PCV), are formed through the coalescence of angioblasts in a process termed vasculogenesis. Around 23 h post-fertilisation (hpf), ECs sprout from the DA to form the primary ISVs, which are consequently all arterial by origin. These primary ISVs fuse at the dorsal side of the trunk to form the dorsal longitudinal anastomotic vessel (DLAV). Around 30-32 hpf, a second wave of sprouting occurs, this time originating from the PCV. These secondary sprouts either form a stable connection to a primary ISV, remodelling it into a venous ISV following its disconnection from the DA, or sprout to the level of the horizontal myoseptum to contribute to lymphatic structures (Kuchler et al., 2006; Isogai et al., 2003; Geudens et al., 2010; Yaniv et al., 2006). This process leads to the formation of a balanced network of arteries and veins that efficiently delivers blood throughout the trunk. What drives the outcome of secondary sprouting – vein formation or lymphatic contribution – is still poorly understood. Several publications have suggested that secondary sprouts forming either venous ISVs or lymphatic structures are genetically different when emerging from the PCV (Hogan et al., 2009a; Nicenboim et al., 2015; Koltowska et al., 2015; Geudens et al., 2010; Yaniv et al., 2006). This concept proposes that the ultimate fate of an ISV is determined by the nature of the approaching secondary sprout, i.e. a secondary sprout with a lymphatic fate restriction will lead to arterial maintenance, whereas a secondary sprout lacking lymphangioblast identity will remodel the ISV into a vein. Current concepts favour the idea that secondary sprouts are fate-restricted by their level of expression of the lymphatic determinant Prox1: cells expressing low levels of Prox1 will connect to the primary ISV and form a vein, whereas sprouts expressing high levels continue to the level of the horizontal myoseptum and give rise to the lymphatic precursor structures (Nicenboim et al., 2015; Koltowska et al., 2015). However, how such a deterministic program would establish the arterio-venous balance observed throughout the whole organism remains unclear.

Here, using high-resolution live imaging, advanced cell tracking and computational analytics, we have made a series of discoveries showing that the formation of a balanced trunk vasculature involves an unexpected early heterogeneity in EC behaviour in the seemingly identical primary ISVs, and an adaptive flow-mediated mechanism that fine-tunes the balance of arteries and veins along the trunk. Even before connection of the secondary sprouts and in absence of blood flow, the ECs constituting the primary ISVs show distinct behaviour that is predictive for later arterio-venous patterning, showing that the primary ISVs, rather than the secondary sprouts, are pre-programmed to become either an artery or a vein. In addition, and subsequent to this pre-patterning, a flow-mediated mechanism provides flexibility and adaptation to the system to fine-tune the balance of arteries and veins.

## RESULTS

### The trunk vasculature exhibits a global balance of arteries and veins, and local patterns favouring alternating vessel identities

**Fig. 1.**
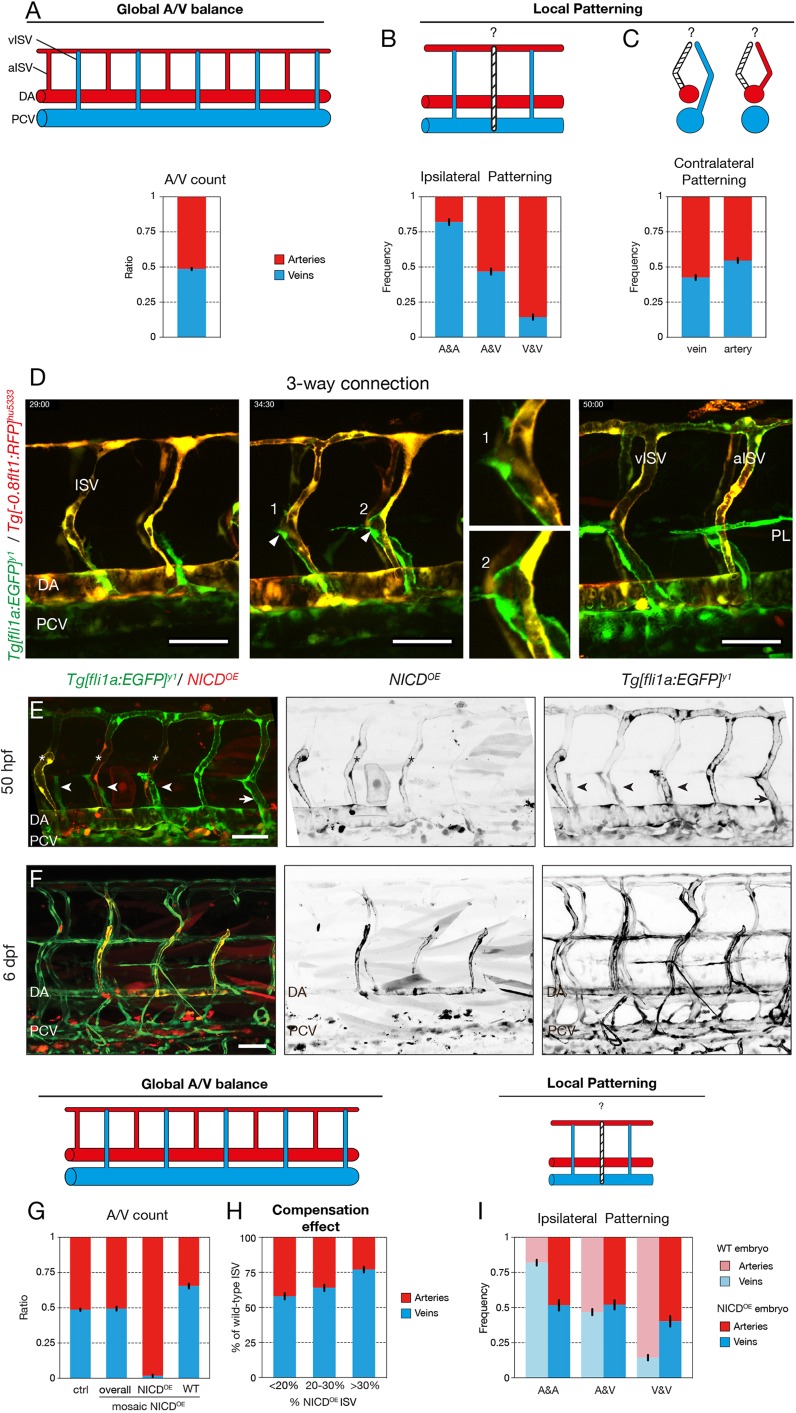
**Notch mediates the local patterning of the trunk vasculature.** (A) Quantification of the ratio of arterial and venous ISVs in a 10-somite region of the trunk of 6 dpf wild-type embryos (*n*=3 experiments, 74 embryos, 1480 ISVs). (B) Ipsilateral neighbourhood analysis of vessel identity with two neighbours in 6 dpf wild-type embryos (*n*=3 experiments, 74 embryos, 1184 ISVs). (C) Contralateral neighbourhood analysis of vessel identity in 6 dpf wild-type embryos (*n*=3 experiments, 74 embryos, 1480 ISVs). (D) Stills from time-lapse movie (Movie 1) of a *Tg[fli1a:EGFP]^y1^/Tg[-0.8flt1:RFP]^hu5333^* embryo showing ISV remodelling into a venous (left) and an arterial (right) intersegmental vessel (vISV and aISV). The double transgenic labelling with GFP expression in all ECs and RFP expression in arterial ECs facilitates distinction between arterial (yellow) and venous (green) structures. In both cases, a lumenised connection is formed between the secondary sprout and the primary ISV (arrowheads). Magnifications show lumenised connections in a future vISV (1) and a future aISV (2). In the case of the formation of an aISV, the connection is lost again and the secondary sprouts form lymphatic precursors at the horizontal myoseptum (parachordal lymphangioblasts, PL). In case of vISV remodelling, the secondary sprout connection is stabilised and the connection between primary ISV and DA regresses. (E) *Tg[fli1a:EGFP]^y1^* embryos mosaically expressing a *pT2Fli1ep-zN1aICD-basfli-mCherry* construct (NICD^OE^) at 50 hpf. Lymphangiogenic sprouts, i.e. sprouts delivering lymphatic precursors at the horizontal myoseptum (arrowheads), can be observed at the position of NICD overexpressing (NICD^OE^) ISVs (asterisks). Arrow points to a venous ISV connection. (F) *Tg[fli1a:EGFP]^y1^* embryos mosaically expressing a *pT2Fli1ep-zN1aICD-basfli-mCherry* construct at 6 dpf. NICD^OE^ mCherry-positive cells were found almost exclusively in the arterial compartment of the vasculature. (G) Quantification of the ratio of arterial and venous ISVs in a 10-somite trunk region of 6 dpf control embryos (*n*=3 experiments, 74 embryos) and mosaic NICD^OE^ embryos (*n*=3 experiments, 51 embryos). In mosaic embryos, the arterio-venous distribution was quantified overall and separately in NICD^OE^ and wild-type ISVs. Wild-type ISVs compensate for the forced arterialisation of NICD^OE^ ISVs by increased formation of venous connection. (H) Quantification of the percentile presence of arterial and venous ISVs in a 10-somite region of the trunk of 6 dpf NICD^OE^ embryos. Mosaic NICD^OE^ embryos represented in G were grouped based on their relative number of NICD^OE^ ISVs (<20%, 20-30% or >30%; *n*=13, 26 and 12 embryos, respectively). (I) Ipsilateral neighbourhood analysis of vessel identity with two neighbours in 6 dpf NICD^OE^ embryos (*n*=3 experiments, 40 embryos, 592 ISVs) compared with wild-type embryos (*n*=3 experiments, 74 embryos, 1184 ISVs). A, artery; DA, dorsal aorta; PCV, posterior cardinal vein; ISV, intersegmental vessel; PL, parachordal lymphangioblasts; V, vein; WT, wild type; NICD^OE^, NICD overexpressing. Scale bars: 50 μm.

The zebrafish trunk vasculature consists of a balanced network of arteries and veins (Fig. 1A) (Bussmann et al., 2010). To explore the nature of this global artery-vein balance, we analysed the sequence of arteries and veins on both sides of a 10-somite segment in 6 days post fertilisation (dpf) wild-type embryos, in which arteries and veins are already functionally specified. Performing a neighbourhood analysis to determine the conditional probabilities of forming an artery or a vein given the status of the neighbouring vessels uncovered a strong, albeit imperfect, ipsilateral patterning of alternating vessel fates (Fig. 1B, Fig. S1A,B). Contralateral patterning between opposing vessels in the zebrafish trunk is less pronounced, providing only a weak prediction of vessel identity (Fig. 1C). Taken together, an ISV surrounded by arterial ISVs (aISV) has a high probability of being a venous ISV (vISV).

### Secondary sprouts emerging from the PCV commonly establish a connection with primary ISVs regardless of their future identity

Previous studies identified an early patterning event that occurs in the PCV, establishing either venous or lymphatic cell fate in cells forming the secondary sprouts (Koltowska et al., 2015; Nicenboim et al., 2015). The resulting fate restriction is thought to determine whether the secondary sprout will connect to the primary ISV to form a vein, or will not connect and instead form lymphatic structures. Surprisingly, we found regular interactions between the primary ISV and the secondary sprout irrespective of the final outcome of the patterning event (Fig. 1D; Movie 1). In most segments, secondary sprouts fuse with the primary ISV, forming three-way connections between the DA, ISV and PCV. Ultimately, however, the connection to the DA either regresses, turning the ISV into a vein, or remains stable, thus preserving arterial ISV identity. In the latter case, the secondary sprout disconnects again from the ISV and contributes to lymphatic formation (Fig. 1D). Occasional occurrence of these three-way connections has previously been reported, albeit in only 3% of the ISVs (Isogai et al., 2003). Interestingly, our quantification reveals that at least 77.5% of future aISVs (40 aISVs, *n*=43 embryos) are transiently connected to secondary sprouts, forming a lumenised and perfused shunt (Fig. S1C, Movie 2).

### Notch mediates local patterning of the trunk vasculature

Because of the known role of Notch in regulating artery-vein specification (Lawson et al., 2001; Lawson and Weinstein, 2002; Zhong et al., 2001, 2000) and the increased formation of vISVs upon Notch inhibition (Geudens et al., 2010; Hogan et al., 2009b), we asked whether Notch activity cell-autonomously influences ISV specification. We used Tol2 transgenesis to mosaically overexpress the intracellular domain of Notch1a (NICD) together with an mCherry reporter in single ECs of embryos expressing the vascular reporter *Tg[fli1a:EGFP]^y1^* (Fig. S1D). At 6 dpf, mCherry-positive NICD-overexpressing cells were found almost exclusively in the arterial compartment of the trunk vasculature (DA and aISVs) (Fig. 1F,G), indicating that Notch signalling might play a cell-autonomous role in ISV patterning. Overexpressing the intracellular domain of the paralogue Notch1b (N1bICD) and a dominant-active version of Suppressor of Hairless [Su(H)], the transcription factor mediating canonical Notch signalling, confirmed this idea (Fig. S1E). Live imaging revealed that the ISV containing NICD-overexpressing cells remained arterial, while still forming transient lumenised three-way connections (Fig. S1F, Movie 3). Interestingly, when studying all vessels in these embryos, we observed a strong bias towards the formation of veins in ISVs containing only wild-type cells (Fig. 1G). Moreover, when analysing individual embryos, we observed that with increasing numbers of ISVs containing NICD-overexpressing cells, proportionately more wild-type ISVs turned into veins (Fig. 1H). As a result, embryos with mosaic overexpression of NICD maintained the global artery-vein balance (Fig. 1G). Neighbourhood analysis in these embryos showed a complete disruption of ipsilateral patterning, rendering all local patterns equally probable (Fig. 1I). Taken together, these results demonstrate a cell-autonomous role for high Notch activity in locally instructing artery formation, and suggest the existence of a compensation mechanism that maintains the global balance between arteries and veins, independently of the local patterning.

### Flow mediates the global artery-vein balance of the trunk vasculature

**Fig. 2.**
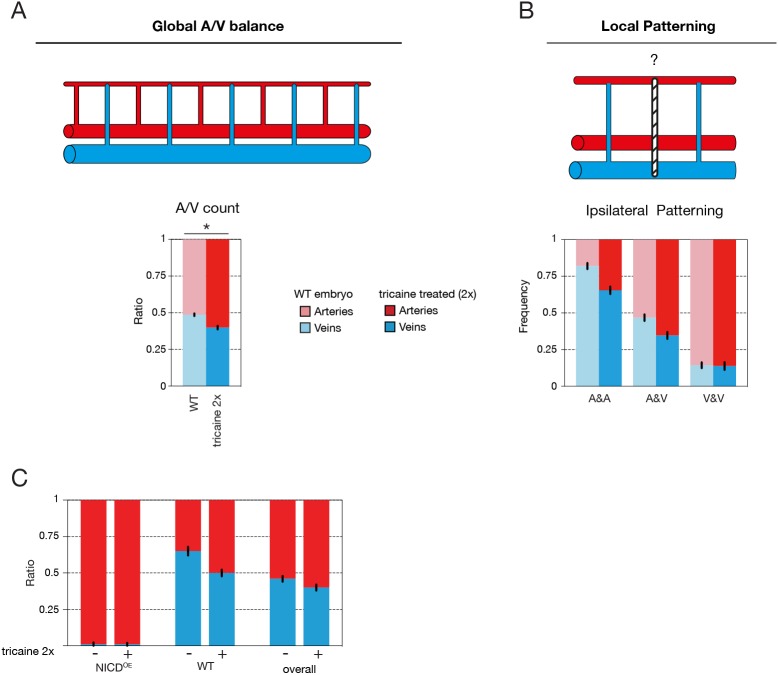
**Flow mediates the global artery-vein balance of the trunk vasculature.** (A) Quantification of the ratio of arterial and venous ISVs in a 10-somite region in the trunk of 6 dpf wild-type embryos either untreated (control; *n*=3 experiments, 74 embryos) or treated with 2×tricaine to slow down the heart rate (*n*=3 experiments, 65 embryos) (*P<0.0001, two-tailed unpaired *t*-test). (B) Ipsilateral neighbourhood analysis of vessel identity with two neighbours in 6 dpf wild-type embryos either untreated (control; *n*=3 experiments, 74 embryos, 1184 ISVs) or treated with tricaine to slow down the heart rate (*n*=3 experiments, 65 embryos, 950 ISVs). (C) Quantification of the ratio of arterial and venous ISVs in a 10-somite trunk region of 6 dpf mosaic NICD^OE^ embryos either untreated (−) or treated (+) with 2×tricaine to slow down the heart rate (respectively: *n*=3 experiments, 51 embryos; *n*=2 experiments, 20 embryos). In mosaic embryos, the arterio-venous distribution was quantified overall and separately in mosaic NICD^OE^ and wild-type ISVs (WT). Blood flow reduction eliminates the compensating venous remodelling in wild-type ISVs in mosaic NICD^OE^ embryos, resulting in and overall shift towards more arterial ISVs.

As a balanced artery-vein network is required for optimal blood flow distribution in the fish trunk, we speculated that flow and flow sensing play an important role in the patterning and/or compensation of vessel specification. To test this hypothesis, we slowed down the heart rate by treating embryos with tricaine (tricaine mesylate, MS-222), a muscle relaxant commonly used as an anaesthetic in fish. Treatment of the embryos with twice the dose normally used for anaesthesia, after the onset of secondary sprouting, from 31 to 52 hpf, significantly reduced heart rate and blood flow speed (Fig. 2). Flow reduction in wild-type embryos disrupted the global balance of arteries and veins (61.2±0.06 % aISV) (Fig. 2A) while retaining ipsilateral patterning (Fig. 2B). Accordingly, blood flow could be a crucial determinant in the compensation mechanism that establishes the overall balance in the number of arteries and veins along the trunk. Indeed, treating embryos harbouring mosaic overexpression of NICD in the vasculature with a similar dose of tricaine resulted in a reduction in the number of wild-type vessels becoming vISVs, thus abolishing the compensation effect (Fig. 2C).

### Differential polarity and directional movement of ECs predict arterial or venous fate

**Fig. 3.**
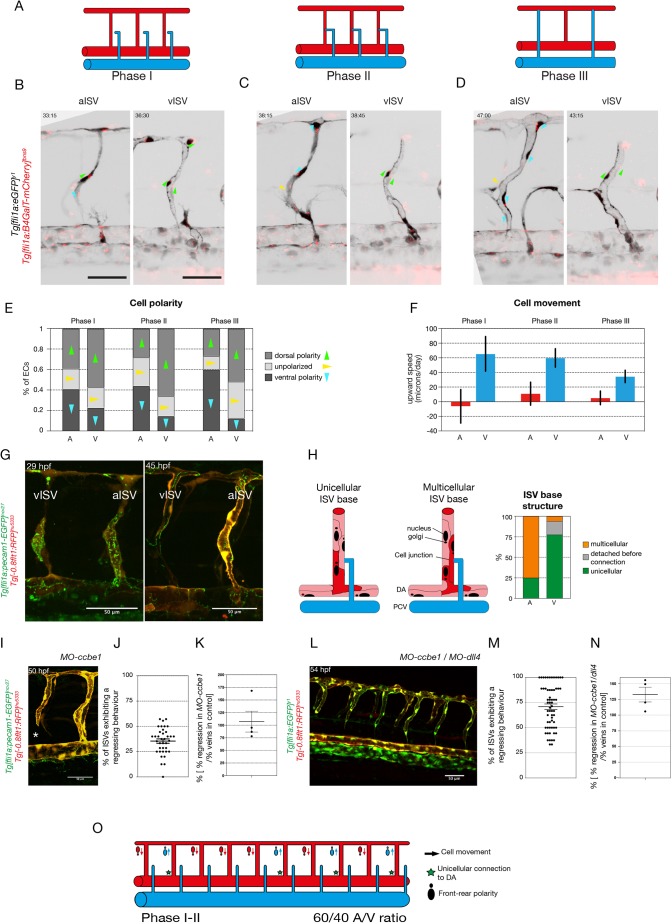
**Primary ISVs are specified into aISVs and vISVs prior to connection by secondary sprouts originating from the PCV.** (A) Schematic representation of the three phases of primary ISV remodelling: phase I, before secondary sprout connection to the primary ISV; phase II, when a lumenised connection is formed between the secondary sprout and the primary ISV; phase III, when the three-way connection resolves into aISV or vISV. (B-D) Stills from time-lapse movies (see Movies 4 and 5) of EC polarity in aISVs and vISVs of *Tg[fli1a:GFP]^y1^;Tg[fli1a:B4GalT-mCherry]^bns9^* embryos during the three different phases: (I) before secondary sprout connection, (II) during three-way connection and (III) after resolution. Arrowheads indicate the angle from the centre of the nucleus to the centre of the Golgi complex: green indicate dorsal polarity; blue indicate ventral polarity; yellow indicate unpolarised ECs. (E) Quantification of EC polarity in aISVs (*n*=7 aISVs, 16 cells) and vISVs (*n*=8 vISVs, 17 cells) of *Tg[fli1a:GFP]^y1^;Tg[fli1a:B4GalT-mCherry]^bns9^* embryos during the three different phases: (I) 2.5 h before secondary sprout connection; (II) during three-way connection; and (III) 2.5 h after resolution. (F) Quantification of EC upward speed (in microns/day) in aISVs (*n*= 12 aISVs, 67 cells) and vISVs (*n*=13 vISVs, 103 cells) during the three different phases: (I) 1 h before secondary sprout connection; (II) during three-way connection; and (III) 1 h after resolution. (G) Stills from time-lapse movie (Movie 6) of a *Tg[fli1a:pecam1-EGFP]^ncv27^;Tg[-0.8flt1:RFP]^hu5333^* embryo showing ISV remodelling into an arterial and a venous intersegmental vessel (aISV and vISV) at 29 and 45 hpf. (H) Quantification of the cellular structure at the base of the primary ISV at the inception of phase II. Detection or absence of GFP expression is used to characterise the nature of the connection (unicellular or multicellular) (*n*=34 embryos, 12 aISVs, 40 vISVs). (I) Still from a time lapse movie (see Movie 7) of a T*g[fli1a:pecam1-EGFP]^ncv27^; Tg[-0.8flt1:RFP]^hu5333^* 5 ng *MO-ccbe1* embryo showing ISV regression in the absence of secondary sprouting. (J) Quantification of the percentage of primary ISVs exhibiting a regression behaviour (full disconnection from the DA, thin membrane connection to the DA, lumen collapse and reconnection, and cell death at the base of the primary ISV; see Fig. S3) (*n*=4 experiments, 37 morphants, 241 morphant vessels). (K) Quantification of percentage of primary ISVs exhibiting a regression behaviour in *ccbe1* morphants compared with the percentage of veins in control clutch mates (*n*=37 morphants, *n*=29 wild-type controls). (L) Stills from time-lapse movie (Movie 8) of a *Tg[fli1a:EGFP]^y1^;Tg[-0.8flt1:RFP]^hu5333^ 5 ng MO-ccbe1/10 ng MO-dll4* embryo showing ISV regression in the absence of secondary sprouting. (M) Quantification of percentage of primary ISV exhibiting a regression behaviour (full disconnection from the DA, thin membrane connection to the DA, lumen collapse and reconnection, and cell death at the base of the primary ISV; see Fig. S2) (*n*=7 experiments, 62 morphants, 531 morphant vessels). (N) Quantification of percentage of primary ISVs exhibiting a regression behaviour in *MO-ccbe1(5 ng)/MO-dll4 (10 ng)* double morphants compared with the percentage of veins in control clutch mates (*n*=62 morphants, *n*=17 wild-type controls). (O) Schematic representation of ISV specification prior to and at the inception of the three-way connection, quantifiable through EC polarity, upward movement speed and cellular structure at the connection to the dorsal aorta. A, aISV; V, vISV. Scale bars: 50 μm. In J,K,M,N, data are mean± s.e.m. with individual data points indicated.

Given the prevalence of three-way connections as an intermediate step in remodelling, the issue of whether a given ISV remains arterial or becomes a vein is determined by which of the branches of the three-way connection is stabilised or lost. This is reminiscent of the pruning process in capillary networks where directional migration of cells drives regression of poorly perfused vessel segments (Chen et al., 2012; Franco et al., 2015). Previous studies of developmental vessel pruning in mouse retina and zebrafish brain established that ECs exposed to physiologically high levels of blood flow, above a certain threshold, align in the direction of the flow and polarise against it (Franco et al., 2015; Tzima et al., 2005). As a consequence, adjacent vessel segments (branches) that experience different levels of flow show opposite endothelial polarisation and movement. ECs polarise and migrate away from the branch, experiencing low or sub-threshold flow, into the branch under high flow. The divergent polarity of cells in the low-flow branch causes cells to disconnect. Therefore, we analysed the polarity of ECs during the remodelling process in *Tg[fli1a:GFP]^y1^;Tg[fli1a:B4GalT-mCherry]^bns9^* embryos, labelling ECs in green and the endothelial Golgi apparatus in red. We analysed polarity at three different phases: (I) before secondary sprout connection; (II) when the three-way connection is present; and (III) after resolution (Fig. 3A, Fig. S4A). We found that most ECs polarise against the flow, leading to ventral polarity in aISVs [60% (105/175) of ECs] and dorsal polarity in vISVs [52% (71/137) of ECs] after remodelling (phase III) (Fig. 3B-E, Movies 4 and 5). Surprisingly, ECs in future aISVs and vISVs already showed differential polarity before secondary sprout connection (Fig. 3E – phase I). Moreover, we tracked the nucleus of ECs in all three phases in double transgenic *Tg[-0.8flt1:RFP]^hu5333^*;*Tg[fli1a:EGFP]^y1^* embryos that allow clear distinction of arteries (labelled in green and red) and veins (labelled only in green). We observed that cells in the primary ISV moved dorsally in future vISVs, whereas cells in future aISVs remained largely at the same position or moved slightly ventrally (Fig. 3F). Again, this difference was already present in phase I, illustrating that ECs within the primary ISVs forming future arteries or veins behave differently early on, before any interactions with the secondary sprouts from the PCV take place.

As vessel regression events are characterised by a progressive conversion from multicellular to unicellular arrangements (Franco et al., 2015; Lenard et al., 2015), we analysed the remodelling process by imaging cellular junctions at the base of the primary ISV in *Tg[fli1a:pecam1-EGFP]^ncv27^;Tg[-0.8flt1:RFP]^hu5333^* (junctions in green, arterial structures in red) embryos during remodelling. For most future vISVs, at the moment of connection to the secondary sprout, the base of the ISV was made of a single EC connecting the vessel to the DA. In contrast, the majority of future aISVs had a multicellular arrangement at the ISV base (Fig. 3G,H, Movie 6). Combined, these findings uncover a heterogeneity in primary ISVs, showing differential behaviour of ECs that is predictive of their specification, prior to the connection with secondary sprouts (Fig. 3N). These findings also suggest the possibility that the process of disconnecting from the DA to form a vISV may be initiated independently of the approaching secondary sprout.

### Primary ISV pre-patterning occurs independently of secondary sprouts

To test this hypothesis, we inhibited the formation of secondary sprouts by inactivating *ccbe1*, a crucial mediator of Vegfc processing (Hogan et al., 2009a). Despite the absence of secondary sprouts in *ccbe1* morpholino-treated embryos, a subset of ISVs (on average 38.2±4.8%) showed a dynamic behaviour consistent with regression of the DA connection (Fig. 3I-K, Movie 7). Regressing behaviour was evident by the presence of only a thin membrane connection, lumen collapse and reconnection, and even full detachment of the ISV from the DA in 36.6% (±11.32%) of regressing ISVs (Fig. S3A,B). The regressing ECs exhibited dorsal polarity and movement, a behaviour consistent with venous specification (Fig. S4C,D). Interestingly, co-injection with a *dll4* morpholino, which leads to the formation of an increased number of veins in the trunk (Leslie et al., 2007), resulted in a dramatic increase of ISV regression (*MO-dll4*+*MO-ccbe1*: 70.9±21.54%) (Fig. 3K,L, Fig. S3C, Movie 8). Together, these results suggest that the autonomous regression behaviour observed in the absence of secondary sprouts is associated with a venous specification that is established early on in primary ISVs.

### The majority of primary ISVs are not perfused before secondary sprouts connection

**Fig. 4.**
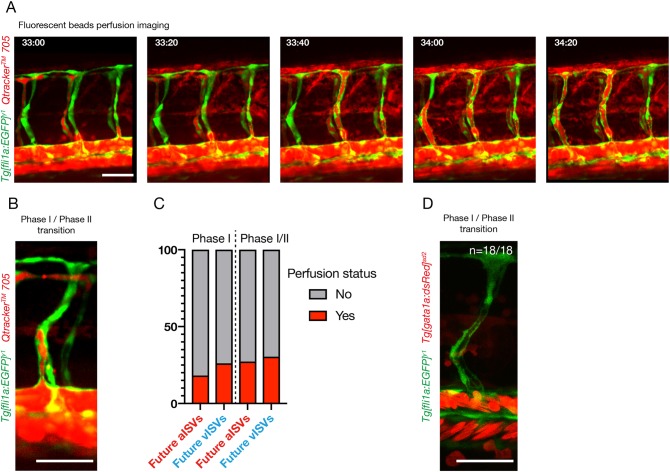
**The majority of primary ISVs are not perfused prior to connection to the secondary sprouts originated from the PCV.** (A) Stills from time-lapse movie of the trunk region of a *Tg[fli1a:EGFP]^y1^* embryo showing perfusion with Qtracker 705 quantum dots between 33 and 34:20 hpf. (B) Stills from time-lapse movie of a *Tg[fli1a:EGFP]^y1^* embryo at time of connection of a primary ISV to a secondary sprout (transition from phase I to phase II) injected with Qtracker 705 quantum dots 705 fluorescent beads. (C) Quantification of primary ISV perfusion in phase I and at time of connection to the secondary sprout (phase I/phase II transition). Phase I perfusion is quantified 1 h before connection to the secondary sprout. Perfusion is defined by the continuous labelling of the lumen area ISV with the quantum dots and visible presence of a probable inlet and outlet for flow (*n*=13 embryos, 23 vISVs, 11 aISVs). (D) Stills from time-lapse movie of a *Tg[fli1a:EGFP]^y^, Tg[gata1a:dsRed]^sd2^* embryo (labelling ECs in green and blood cells in red) at time of connection of a primary ISV to a secondary sprout (transition from phase I to phase II) (representative of *n*=7 embryos, 7 aISVs, 11 vISVs). Scale bars: 50 μm.

To assess whether flow could play a role in the specification of primary ISV prior to phase II, we investigated the perfusion status of these vessels. At the end of phase I/inception of phase II, 25% (3/12) of future vISVs and 11% (1/9) of future aISVs were lumenised throughout (from the DA to the DLAV) (*n*=3 experiments, 7 zebrafish embryos). However, none appeared to exhibit full ventral-to-dorsal lumenisation prior to the connection to the secondary sprout. As lumenisation itself is not sufficient to assess perfusion (as defined by the transit of blood through the ISV), we investigated perfusion both with erythrocytes and with serum. To assess perfusion with serum, we injected *Tg[fli1a:EGFP]^y1^* embryos with fluorescent beads in the general circulation (Qtracker 705 quantum dots) after the initiation of general blood circulation (Fig. 4A,B) (Martin et al., 2013). We found that 27.3% (3/11) of future aISVs and 30.4% (7/23) of future vISVs showed perfusion with the beads (as characterised by the presence of a continuous detection of the fluorescent beads throughout the primary ISV, from the dorsal aorta to a putative outlet in an adjacent ISV) at the end of phase I/inception of phase II (connection of the primary ISV to the secondary sprout). When analysed 1 h earlier, in phase I, only 18.2% (2/11) of aISVs and 26.1% (6/23) of vISVs showed serum perfusion (Fig. 4C) (*n*=4 experiments, 13 embryos). In *Tg[gata1a:dsRed]^sd2^;Tg[fli1a:EGFP]^y1^* embryos, we were unable to detect any red-labelled erythrocytes transiting through the future aISV (*n*=7) or vISVs (*n*=11) prior to, or at the inception of, phase II (Fig. 4D) (*n*=7 embryos).

Overall, our analysis suggests that the blood perfusion status of the primary ISV prior to the connection to the secondary sprout is unlikely to significantly influence the specification of primary ISVs in phase I. In addition, these results support the idea that polarisation of ECs during phase I (see Fig. 3) precedes the polarisation against the direction of flow.

### Notch signalling mediates early primary ISV specification

**Fig. 5.**
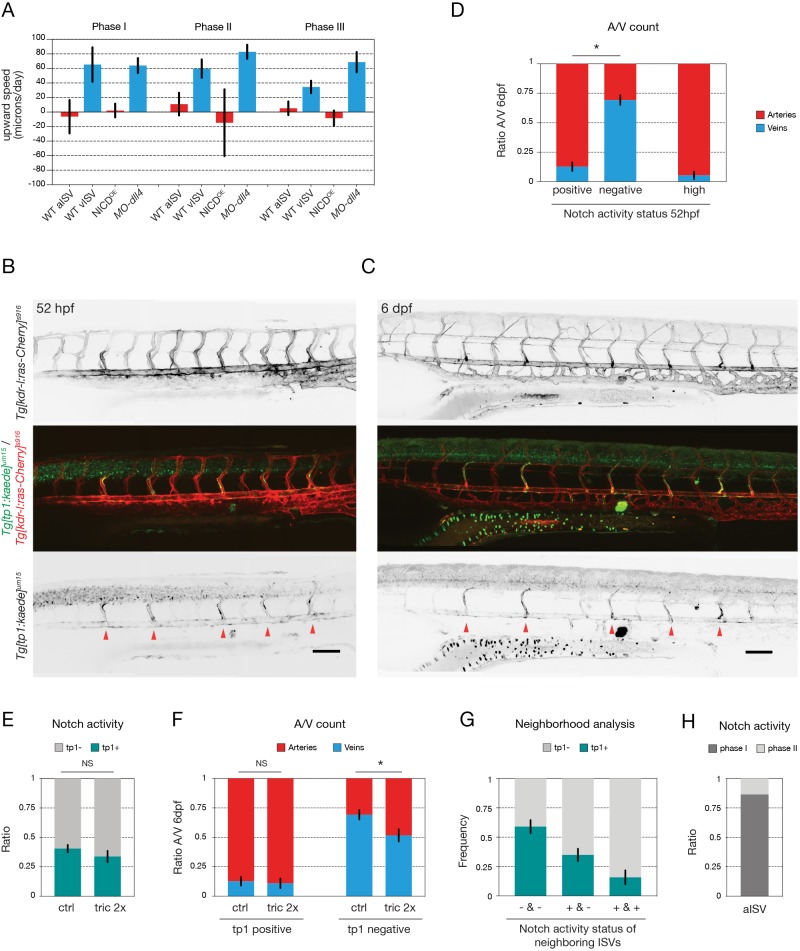
**Notch signalling mediates early primary ISV specification.** (A) Quantification of ECs upward speed (in microns/day) in ISVs of wild-type [*n*=12 aISV (67 cells), 13 vISV (103 cells)], NICD^OE^ (*n*=30 aISV, 29 NICD^OE^ cells) and *MO-dll4* (*n*=9 vISV, 85 cells) embryos (32 to 54 hpf) at three different time points: (I) 2.5 h before secondary sprout connection; (II) during three-way connection; and (III) 2.5 h after resolution of the three-way connection. (B,C) Notch activity reporter *Tg[tp1-MmHbb:kaede]^um15^*;*Tg[kdr-l:ras-Cherry]^s916^* imaged at 52 hpf (B) and at 6 dpf (C) in the same embryo, after conversion of the Kaede photoconvertible fluorescent protein at 29 hpf (Notch activity reporter shown in green, all ECs labelled in red). Red arrowheads indicate ISVs expressing high levels of the Kaede^green^ protein, both at 52 hpf and at 6 dpf. (D) Quantification of the ratio of arteries and veins, determined at 6 dpf, correlated to the Notch activity status at 52 hpf, after conversion of the Kaede photoconvertible fluorescent protein at 29 hpf [tp1 positive, negative or high (as indicated by red arrowheads in B)] (*n*=20 embryos; *P<0.0001, two-way ANOVA). (E) Quantification of the percentage of tp1-positive (tp1+) and tp1-negative (tp1−) ISVs in untreated (*n*=20 embryos) versus 2×tricaine-treated (tric 2×) embryos (*n*=12 embryos) at 52 hpf, after conversion of the Kaede photoconvertible fluorescent protein at 29 hpf. Flow inhibition between 29 and 52 hpf does not affect tp1 promoter activity during this period. (F) Quantification of the ratio of arteries and veins, determined at 6 dpf, correlated to the Notch activity status at 52 hpf (tp1 positive or tp1 negative), in untreated (*n*=20 embryos) versus 2×tricaine-treated embryos (*n*=12 embryos). Flow inhibition does not affect the balance of arteries and veins formed from tp1-positive ISVs, but tp1-negative ISVs form significantly more arteries after tricaine treatment (*P=0.027, two-way ANOVA). (G) Ipsilateral neighbourhood analysis of Notch activity status of vessels with two neighbours in 52 hpf embryos after conversion of the photoconvertible Kaede protein at 29 hpf. Graph shows the frequency of finding a tp1-positive or -negative ISV, given the Notch activity status of the neighbouring ISVs [tp1 positive (+) or tp1 negative (−)] (*n*=19 embryos, 199 ISVs) (*P<0.0001, one-way ANOVA). (H) Quantification of the ratio of arterial ISVs with activated Notch signalling first detectable (as indicated by tp1 signal) before connection of the secondary sprout (i.e. during phase I) or after connection of the secondary sprout (i.e. during phase II) (*n*=22 ISVs). Data are mean±s.e.m. NS, not significant. Scale bars: 100 μm.

Given the strong influence of Notch activity on primary ISV specification, we analysed the effect of manipulating Notch signalling on EC behaviour within the ISV. Tracking of cell movement in *dll4* morphants showed that the primary ISV cells migrated dorsally like wild-type venous cells, whereas in ISVs overexpressing NICD, ECs instead migrated ventrally like arterial cells. Both behaviours are visible in all phases of remodelling (Fig. 5A). Analysis of cell polarity during remodelling in *dll4* morphant embryos showed that the majority of cells are polarised dorsally, as in wild-type vISVs, but that an increased number of cells appear unpolarised (Fig. S4E). Similarly, in flow-chamber experiments under shear stress conditions that mimic physiological flow, we found that chemical inhibition of Notch signalling using the gamma-secretase inhibitor DAPT prevented HUVECs from efficiently orienting parallel to the flow and from polarising against the flow (Fig. S4F).

Thus, our combined *in vivo* and *in vitro* results indicate that Notch signalling influences EC polarity and movement in ways that are predictive of vessel specification into future arteries. However, such a mechanism of artery-vein specification would imply that endogenous Notch activity should be heterogenous early on in primary ISVs.

To analyse Notch activity in wild-type embryos during phase II we used a Notch activity reporter line based on the Epstein-Barr virus tp1 enhancer containing 12 concatamers of Su(H)/Rbpj-binding sites and a minimal promoter (Parsons et al., 2009). To avoid misinterpretation due to time delays in protein degradation, we turned to a photoconvertible version of the Notch activity reporter *Tg[tp1-MmHbb:kaede]^um15^* (Clements et al., 2011) and crossed it to *Tg[kdr-l:ras-Cherry]^s916^* to visualise blood vessels. The photoconvertible fluorescent protein Kaede was converted from Kaede^green^ into Kaede^red^ protein by UV exposure at 29-30 hpf, a time-point before the start of secondary sprouting. This allowed us to analyze the generation of newly formed Kaede^green^ protein, indicating still active Notch signalling. The embryos were imaged at 52 hpf and again at 6dpf to determine the Notch activity status during remodelling and the final artery-vein sequence, respectively. This experiment clearly revealed an early heterogeneity in Notch signalling in the primary ISVs, before and during connection of the secondary sprouts, with multiple ECs within the ISV being labelled in a majority of cases (2.4±1.1 cells/ISV; *n*=31 ISVs) (Fig. 5B, Movie 9). Active Notch signalling during phase II of vascular remodelling correlated significantly with arterial specification (87.3% ±16.4 aISV; *n*=20 embryos), whereas the absence of Notch activity during phase II correlated with venous specification (69.2% ±18.3 vISV; *n*=20 embryos) (Fig. 5D). Of note, we also observed that some ISVs expressed higher levels of Kaede^green^ than others, indicating stronger Notch activity, correlating even more strongly with arterial development (94.7% ±13.3 aISV; *n*=18 embryos) (Fig. 5B-D). These high Notch activity ISVs were often found in alternating positions with lower Notch activity ISVs (Fig. 5B,C, red arrows), corresponding with the observed local patterning favouring alternating vessel identities (Fig. 1B). Indeed, neighbourhood analysis determining the conditional probability of finding a Notch-activated vessel (i.e. tp1 positive) given the Notch activity status of its neighbours showed that tp1-positive vessels are more frequently flanked by tp1-negative vessels (Fig. 5G). This effect was even more pronounced for the high Notch activity ISVs (not shown).

Given the effect of flow inhibition on the global artery-vein balance, we investigated the effect of flow inhibition on Notch activity in embryos with a shifted artery-vein balance. After conversion of the Kaede protein, embryos were treated with 2× tricaine from 30-52 hpf (during phase II). Again, the embryos were imaged at 52 hpf and 6 dpf. The overall percentage of ISVs with active Notch signalling did not change after flow inhibition (40.5±14.2% versus 33.8±16.7%, *n*=20 control versus 12 tricaine-treated embryos, *P*>0.99) (Fig. 5E). In addition, the arterial specification of ISVs with active Notch signalling was not affected (87.3±16.4% versus 89.0±14.2%, *n*=20 control versus 12 tricaine-treated embryos, *P*=0.99) (Fig. 5F). However, we found that ISVs negative for the tp1-reporter formed significantly more arteries in flow-inhibited embryos than in untreated control embryos (30.8±18.3% versus 48.3±17.9%, *n*=20 control versus 12 tricaine-treated embryos, *P*=0.026) (Fig. 5F). Finally, and crucially, we show that, in the majority of tp1-positive future aISVs (86.4%, *n*=22), the Notch pathway is activated before connection of the secondary sprout (Fig. 5H, Movie 10). Taken together, these results indicate that Notch signalling is an important determinant for arterial specification, whereas blood flow is required to fine-tune the global artery-vein balance.

### DISCUSSION

Initial formation of the stereotyped primary vascular network in the zebrafish trunk has been extensively studied (Lawson and Weinstein, 2002; Isogai et al., 2003; Jin et al., 2005; Blum et al., 2008; Herbert et al., 2009). However, the mechanisms through which the trunk vasculature remodels into a balanced network of arteries and veins, and especially how primary ISV either remain arterial or remodel into veins, is less well understood.

An important research focus has been the heterogeneous nature of secondary vascular sprouts emerging from the PCV. Recent work has shown that *prox1* expression levels in the nascent secondary sprouts can be correlated with their future specification as lymphatic endothelial cells (LECs) or venous ECs (Nicenboim et al., 2015; Koltowska et al., 2015). Accordingly, lymphatic precursors arise from secondary sprouts expressing *prox1a*, whereas the majority of Prox1a-negative sprouts connect to the primary ISVs and contribute to the formation of vISVs (Nicenboim et al., 2015; Koltowska et al., 2015). Although never directly tested, this concept would seem to imply that the issue of whether any given primary ISV will remain arterial or will remodel into a vein is determined by the Prox1a-mediated fate restriction of the secondary sprout it encounters.

**Fig. 6.**
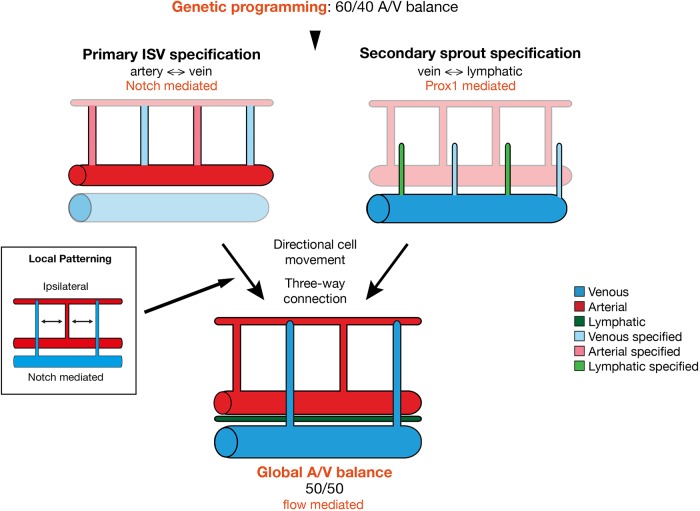
**Alternative mechanism of artery-vein specification in the zebrafish trunk.** We show that genetic programming alone pre-patterns the trunk ISVs to form 60% arteries and 40% veins. This primary ISV specification is largely mediated by Notch signalling that locally instructs artery formation and occurs in a way that favours ipsilaterally alternating vessel identities. Previously it has been shown that lymphovenous specification of the secondary sprouts is mediated by Prox1 signalling (Nicenboim et al, 2015; Koltowska et al, 2015). Upon connection of secondary sprouts to primary ISVs, transient three-way connections are formed that resolve into either arterial or venous ISV connections. This remodelling is governed by a combination of pre-specified and flow-induced directional migration of ECs, ultimately resulting in a global balance of arteries and veins throughout the trunk vasculature.

However, our work demonstrates that the future arterial or venous fate of the ISV can be largely predicted by endothelial heterogeneity in the primary ISVs. Furthermore, this heterogeneity in signalling activity, EC polarity and directional EC movement appears pre-specified prior to the formation of any connection with the approaching secondary sprout. At first glance, these results are therefore incompatible with the idea that vascular remodelling events in the zebrafish trunk are driven by the lymphatic-vein specification of secondary sprouts under the governance of Prox1a activity. However, a more-detailed examination of the remodelling process, the different early specification events, the intermediate steps in remodelling and continuous tracking of cells during the process leads to a picture that may partially reconcile both models (Fig. 6).

Although our present results clearly identify Notch-mediated specification of primary ISV arterio-venous fate through directional movements and junctional configurations that appear to stabilise ISV connections to the DA, and thereby retain arterial identity, they do not necessarily mean that Prox1a levels cannot determine lympho-venous EC fate decisions. Our study identifies that most secondary sprouts engage in a connection with the primary ISV irrespective of any pre-specification of either the primary ISV or the secondary sprout. This would mean that the ultimate fate of ECs to become arterial, venous or lymphatic is not determined by a decision governing with what they initially connect, but instead by a decision that governs from what they will ultimately disconnect. In the case of an arterial ISV, the critical disconnection will need to occur between ISV and secondary sprout. A venous ISV will instead need to disconnect from the DA. Similarly, a lymphatic EC will need to disconnect from the primary ISV in order to form the parachordal lymphangioblasts. The formation of these parachordal lymphangioblasts has generally been seen to occur wherever the ISV remains arterial, linking the acquisition of lymphatic fate in the secondary sprout to the acquisition of arterial fate in the ISV. Closer observations, however, identify additional and alternative behaviour: Even where secondary sprouts have remodelled an ISV into a vein, lymphangioblasts can occasionally be seen to emerge and disconnect from this vein at a later time point (see Movies 1 and 5). This phenomenon has already been described by Isogai and colleagues in their landmark study (Isogai et al., 2003). Thus, a secondary sprout can give rise to both venous and lymphatic structures, suggesting that the original idea of *prox1a* promoting lymphatic fate may hold true even if the cells intermittently help to form a vein. Such a model would also fit with the observed overabundance of Prox1a high secondary sprouts (Koltowska et al., 2015). Accordingly, 65% of secondary sprouts show high Prox1a expression, demonstrating that some will need to form veins despite their Prox1a expression.

The question of how the overall balance of arteries and veins is established or ‘how do the embryo keeps count’ is therefore not answered by early fate determination events in the secondary sprout. Neither is it determined by the number of Notch high primary ISVs or by the number of vessels that show features of pre-specified arteries. Our quantification points to roughly 60% of vessels showing ventral polarity, and ventral movement, as well as a multicellular junction arrangement at their base. Consequently, this pre-specification would predict that embryos form around 60% arteries and only 40% veins (Fig. 6). Intriguingly, blocking flow leads to numbers very close to this pre-specification, with an overabundance of arteries formed, indicating that flow-mediated compensation induces re-specification of some arteries into veins even in wild-type embryos. A similar 60/40 ratio was observed in artery/vein-specific primary ISVs EC behaviour when blocking secondary sprouting, together suggesting that flow acts on the three-way connection to drive compensation. The extent of this compensation effect appears scalable for larger deviations from the optimal 50/50 ratio, as shown by the mosaic Notch activation experiments, indicating there is no fixed subpopulation of adaptable ISVs. When looking at intrinsic Notch activity, the ISVs containing the highest reporter activity showed almost no deviation from arterial fate no matter whether flow is inhibited or not. However, the Notch low vessels appear to shift to more arteries in the absence of flow (Fig. 5F). Therefore, it is tempting to speculate that ECs showing lower levels of Notch are more plastic and amenable to flow-mediated adaptation.

The issue of how the local pattern favouring alternating arteries and veins is achieved, at least in the ipsilateral analysis, is equally intriguing. Although it would be conceivable that the three-way connection alone coupled to flow-induced polarity can work to favour alternating fates, and thus balance flow distribution, the data suggest something else is at play. Embryos lacking flow maintain local patterning that favours alternating fate, even if the global balance is skewed. Mosaic overexpression of NICD, however, completely disrupted local patterning, suggesting that Notch levels are also part of the local patterning. This idea is supported by the fact that even the early Notch reporter heterogeneity assessed in the *Tg[tp1-MmHbb:kaede]^um15^* line shows local patterning. How this patterning of high and low Notch activity is achieved in the early ISV remains to be studied in future work. Further studies will also need to uncover how flow overwrites the early ISV specification. Previous work suggests that it could do so by directly modulating Notch levels (Watson et al., 2013), although the possibility of a distinct specification pathway cannot be excluded.

Recently, work by Weijts and colleagues (Weijts et al., 2018) has suggested that the artery-vein balance in the zebrafish trunk can be primarily explained by a flow-mediated activation of Notch activity preventing primary ISVs from turning into vISVs. Although we agree on the fact that Notch and flow play a role in artery-vein balance, our models differ significantly, most notably by decoupling the role of Notch and flow during primary ISV specification. Although we also show that flow plays a role in the global artery-vein balance in the trunk vasculature (Fig. 2), our work strongly suggests that primary ISVs are pre-specified in a Notch-dependent process prior to and in the absence of a connection to the secondary sprouts originating from the PCV (Figs 3 and 5). In addition, we show that this pre-specification occurs before the majority of primary ISVs are perfused, suggesting that this process is flow independent (Fig. 4). Finally, we find that inhibition of blood flow with tricaine results in an increased number of arteries (Fig. 2), but not veins, as presented by Weijts et al. (2018). Overall, these results are hard to reconcile with our own.

In conclusion, our work identifies transient artery-vein connections as intermediate structures that resolve through a combination of pre-specified and flow-induced directional migration of ECs. We propose that vascular remodelling, based on a dual mechanism of molecular specification and flow-mediated patterning control, provides the necessary plasticity to allow formation of an overall balanced and efficiently perfused vascular network in the zebrafish trunk.

## MATERIALS AND METHODS

### Zebrafish husbandry and transgenic lines

Zebrafish (*Danio rerio*) were raised and staged as previously described (Kimmel et al., 1995). The following transgenic lines were used: *Tg[fli1a:EGFP]^y1^* (Lawson and Weinstein, 2002) (labels all endothelial cells), *Tg[fli1a:nEGFP]^y7^* (labels all endothelial cell nuclei) (Roman et al., 2002), *Tg[fli1a:dsRedEX]^um13^* (Covassin et al., 2009) (labels all endothelial cells), *Tg[gata1a:dsRed]^sd2^* (Traver et al., 2003) (labels all erythrocytes), *Tg[-0.8flt1:RFP]^hu5333^* (Bussmann et al., 2010) (strongly labels arterial endothelial cells), *Tg[fli1a:B4GalT1-mCherry]^bns9^* (Kwon et al., 2016) (labels the Golgi apparatus of endothelial cells), T*g[fli1a:pecam1-EGFP]^ncv27^* (Ando et al., 2016) (labels the endothelial cell junctions), *Tg[tp1-MmHbb:kaede]^um15^* (Clements et al., 2011) (labels cells with an active Notch signalling pathway) and *Tg[kdr-l:ras-Cherry]^s916^* (Hogan et al., 2009a) (labels endothelial cell membrane). For growing and breeding of transgenic lines, we complied with regulations of the animal ethics committees at KU Leuven and MDC Berlin.

### Vessel patterning analysis

Figs 1A-C,G-J, 2A-C, Figs S1A,B, S2A,B show analyses of the regularity of local and global vessel arrangements of aISVs and vISVs in the zebrafish trunk. To perform those analyses, 6 dpf zebrafish embryos were screened under a fluorescent stereomicroscope (Leica M205 FA) to identify the sequence of arterial and venous ISVs on both flanks of a 10-somite segment in the trunk, starting after the junction of the DA and PCV. The identity of arteries and veins was determined by their connection to, respectively, the DA or the PCV, and by direction of blood flow. This manual characterisation yielded for each vessel its ISV type, its collateral neighbour's ISV type, as well as the ISV type of its left neighbour for all but the leftmost vessels and the ISV type of its right neighbour for all but the rightmost vessels. In mosaic NICD-overexpression experiments, in addition to the vessel type it was also registered whether a given vessel contained a NICD-overexpressing cell (used in Fig. 1H-J). This dataset was then analysed to obtain the global and local distribution patterns of aISVs and vISVs for different experimental conditions using a custom-made Python script. To obtain the global vessel ratio, the number of aISVs and vISVs of all ISVs was compared in Figs 1A,G,H and 2A.

To study the local ISV patterning, the relative frequency of aISVs given the type of the neighbouring vessels was obtained: Figs 1C,J and 2C show the frequency of aISVs given the type of the collateral neighbour (aISV or vISV); Figs 1B,I and 2B show the frequency of aISVs given the corresponding number of ipsilateral aISV neighbours in a vessel (0, 1 or 2 aISVs); Fig. S2A shows the frequency of aISVs for the given number of aISVs in an area of 2 vessels surrounding that vessel (0, 1, 2, 3 or 4 aISVs); and Fig. S1B shows the frequency of aISVs given the type of the right neighbour (aISV or vISV). Fig. 5G similarly shows the frequency of tp1-positive and -negative ISVs given the Notch activity status (tp1 signal) of the ipsilateral neighbours.

### Mosaic overexpression using Tol2 transgenesis

Transgenic zebrafish embryos *Tg[fli1a:EGFP]^y1^* were injected at the one-cell stage with 100 pg of Tol2 mRNA (Kwan et al., 2007) and 15 pg of plasmid DNA *pTol2-N1aICD-basfli-mCherry* (De Bock et al., 2013), 25 pg of plasmid DNA *pTol2-N1bICD-basfli-mCherry* or 40 pg of plasmid DNA *pTol2-Su(H)VP16-basfli-mCherry*. The *pTol2-N1bICD-basfli-mCherry* and *pTol2-Su(H)VP16-basfli-mCherry* constructs were generated by Multisite Gateway cloning (Life Technologies). Embryos were raised at 28°C and screened for transient expression at ∼30 hpf. Quantification of arterial and venous ISV distribution was performed in 6-day-old embryos by scoring their percentile presence in 10 consecutive somite segments in the trunk after the junction of DA and PCV (i.e. somites 5-15).

### Live imaging

Embryos were anaesthetised in 0.014% tricaine (MS-222, Sigma-Aldrich), mounted in a 35 mm glass-bottomed petri dish (0.17 mm, MatTek) using 0.6-1% low melting point agarose (Sigma-Aldrich) containing 0.014% tricaine, and bathed in Danieau's buffer containing 0.007 (0.5×) to 0.014% (1×) tricaine and 0.003% PTU (as indicated). Time-lapse imaging was performed using a Leica TCS SP8 upright microscope with a Leica HCX IRAPO L ×25/0.95 water-dipping objective and heating chamber, or on an upright 3i spinning-disc confocal using a Zeiss Plan-Apochromat 20×, 40× or 63×/1.0 NA water-dipping objective. Image processing was performed using Fiji software (Schindelin et al., 2012).

### Tricaine treatment

To slow down heart rate during the secondary sprouting and ISV remodelling process, embryos were treated with 0.028% (2×) tricaine (MS-222, Sigma-Aldrich) between 31 and 52 hpf, after which the compound was washed away again.

### Cell polarity analysis

To analyze polarity of ECs during vascular remodelling, time-lapse movies were made of transgenic *Tg[fli1a:EGFP]^y1^;Tg[fli1a:B4GalT1-mCherry]^bns9^* embryos during vascular remodelling in the trunk (∼32 hpf to ∼54 hpf). Polarity arrows from the centre of the nucleus to the centre of the Golgi apparatus were drawn manually using Fiji software. For every primary ISV cell, the polarity was scored per time point: dorsal polarity, ventral polarity or unpolarised, depending on the relative position of the Golgi apparatus to the nucleus, i.e. Golgi dorsal, ventral or parallel, respectively, to the nucleus with respect to the local angle of the ISV (Fig. S4A). All scores were added over the different timepoints for each developmental stage, as indicated in the figure legends.

### Cell movement analysis

To analyze the upward movement of ECs within ISVs of *Tg[fli1a:GFP]^y1^* or *Tg[fli1a:nEGFP]^y7^; Tg[fli1a:dsRedEX]^um13^* embryos in different stages of development, we used a mix of manual segmentation of developmental timelapse and computational analysis in Python (Figs 3F,K). Confocal stacks were registered using StackReg (ImageJ plug-in – bigwww.epfl.ch/thevenaz/stackreg/). The distance of the cells to the dorsal aorta was tracked through time in a 2D maximum projection manually in Fiji, and combined with the information about the later fate (aISV or vISV) of the vessel containing the cell, and the current phase of development of the ISV (compare with Fig. 3A). To obtain the upward speed of the cells in the individual phases of vessel development, the initial distance of the nucleus of the cell to the aorta was compared with the final position in the given phase (in μm) divided by the duration of the trajectory of the cell in the phase (in min). Under this definition, a positive upward speed corresponds to an increasing distance from the dorsal aorta (i.e. movement towards the DLAV), whereas a negative upward speed corresponds to an average movement towards the dorsal aorta.

### Perfusion assay

The Qtracker 705 quantum dots solution was injected in the duct of Cuviers of zebrafish embryos anaesthetised with 0.014% tricaine and mountain laterally in 0.0014% low-melting agarose after establishment of blood flow (28 to 30 hpf). The quantum dots were excited with a 561 nm laser and their emission detected between 665.5 and 735.5 nm.

### Morpholino knockdown

Morpholinos against *ccbe1* and *dll4* were used as previously described (Hogan et al., 2009a; Leslie et al., 2007).

### Notch activity reporter assay

For analysis of Notch activity during phase II of vascular remodelling, the Kaede^green^ protein in embryos of the Notch activity reporter *Tg[tp1-MmHbb:kaede]^um15^; Tg[kdr-l:ras-Cherry]^s916^* was converted to Kaede^red^ at 29 hpf by illumination with a 405 nm laser. These embryos were imaged at 52 hpf to assess Notch activity during phase II, i.e. newly formed Kaede^green^ protein. At 6 dpf, the same embryos were imaged again to analyze the artery-vein sequence. For the tricaine experiment, embryos were treated with 0.028% (2×) tricaine from 29 to 52 hpf after conversion of the Kaede protein.

### *In vitro* flow chamber experiments

HUVECs (Promocell, primary cells from pooled donors; characterised by flow cytometry with the following markers CD31^+^, vWF^+^, Dil-Ac-LDL uptake^+^, SMA^−^ and tested for mycoplasma contamination) were seeded and grown to confluency on a slide in EBM2 medium (Promocell) coated with gelatin. Unidirectional laminar shear stress was applied to confluent HUVECs using a parallel plate chamber system (Ramkhelawon et al., 2009) for 24 h and cells were treated with 5μM DAPT or a similar amount of DMSO in controls for the duration of the experiment. Local shear stress was calculated using Poiseuille's law and averaged 20 dyne/cm^2^. Cells were fixed in 100% methanol for 10 min at −20°C and stained for DAPI, VE-cadherin (Santa Cruz Biotechnology, sc-6458, dilution 1/100) and GM130 (BD Biosciences, 610822, dilution 1/400) (Franco et al., 2016). Matlab was used to analyze cell orientation (direction of the main axis of the nucleus) and cell polarity (angle between the centre of the nucleus and the centre of the Golgi).

### Blood flow and heart rate measurements

Embryos were anaesthetised in 0.014% tricaine (MS-222, Sigma-Aldrich), mounted in a 35 mm glass-bottomed petri dish (0.17 mm, MatTek) using 1% low melting point agarose (Sigma-Aldrich) containing 0.007% (0.5×), 0.014% (1×) or 0.028% (2×) tricaine, and bathed in Danieau's buffer containing 0.007% (0.5×), 0.014% (1×) or 0.028% (2×) tricaine, respectively, and 0.003% PTU for 1 h before being imaged on an upright 3i spinning-disc confocal microscope using a Zeiss Plan-Apochromat, 20×/1.0 NA water-dipping objective with a frame interval of 10 ms. Kymographs were generated using the MultipleKymograph plug-in in ImageJ to quantify heart rate over an 8 s period, synced to the beginning of a heartbeat (line width: 1).

To estimate instantaneous blood flow speed, we cropped images of the dorsal aorta and measured average frame-to-frame translation of red blood cells using the Kuglin-Hines algorithm (Kuglin and Hines, 1975) for image phase correlation. In brief, the phase-correlation map between two adjacent frames was calculated by multiplying the Fast Fourier transform (FFt) of frame_i_ and a conjugate FFt of frame_i+1_. The inverse FFt of the phase correlation provides a correlation map with a peak offset from the centre caused by the relative shift between the frames. The position of the peak was determined by finding the local maximum in a Gaussian-filtered correlation map. The velocity data were smoothed with a moving average filter with a span of five frames. Analysis was performed in Matlab (Mathworks).

### Statistical analysis

No statistical method was used to predetermine sample size. Data represent mean±s.e.m. of representative experiments (except when indicated otherwise). Statistical tests were conducted using Prism (GraphPad) software. Adequate tests were chosen according to the data to fulfil test assumptions. Sample sizes, number of repeat experiments, performed tests and *P*-values are indicated per experiment in Table S1. The angle repartitions of the flow chamber experiments were analysed using Kuiper two-sample test, a circular analogue of the Kolmogorov–Smirnov test. *P*<0.05 was considered statistically significant.

Zebrafish embryos were selected on the following pre-established criteria: normal morphology, a beating heart and the presence of circulating red blood cells. The experiments were not randomised. For every experiment, treated and control embryos were derived from the same egg lay. The investigators were not blinded to allocation during experiments and outcome assessment.

## Supplementary Material

Supplementary information
