# Supplementary material for: Artery-vein specification in the zebrafish trunk is pre-patterned by heterogeneous Notch activity and balanced by flow-mediated fine-tuning
Source: Development. 2019 Aug 27;146(16):dev181024. doi: 10.1242/dev.181024 (PMC6737902; doi:10.1242/dev.181024)
Supplement: Supplementary information [file develop-146-181024-s1.pdf]

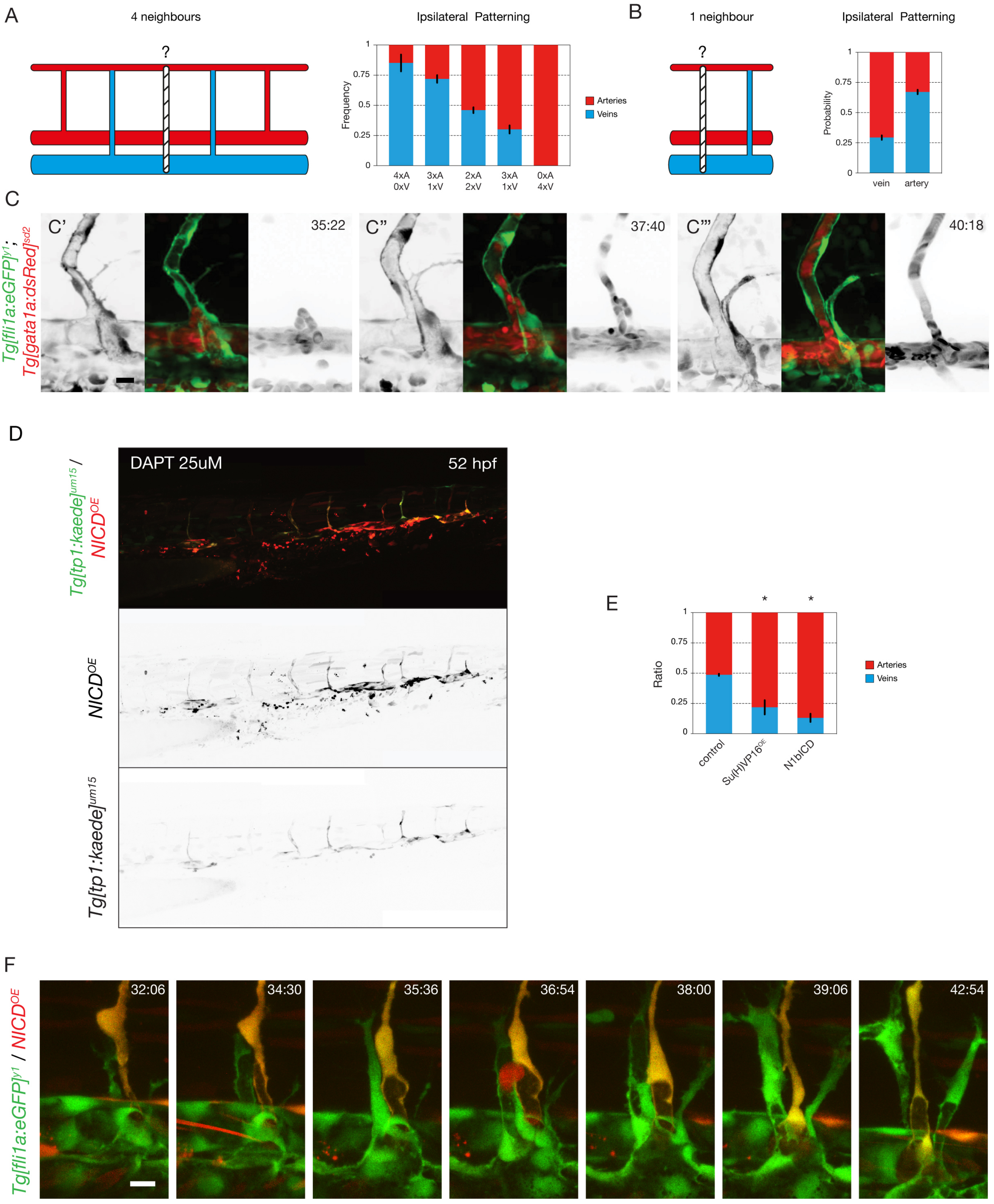


Development • Supplementary information

**Figure S1**

A) Ipsilateral neighborhood analysis of vessel identity with 4 neighbors in 6 dpf WT embryos (N=3 experiments, 74 embryos, 888 ISVs).

B) Ipsilateral neighborhood analysis of vessel identity with 1 neighbor in 6 dpf WT embryos (N=3 experiments, 74 embryos, 1332 ISVs).

C) Stills from time-lapse movie (Supplementary Movie S2) in *Tg[fli1a:GFP]$^{y1}$;Tg[gata1a:DsRed]$^{sd2}$* labeling ECs in green and blood cells in red showing formation of a transient perfused three-way connection (C'-C") as circulating blood cells can be observed in the DA-ISV-secondary sprout-PCV shunt. In panel C"' it is clear that the ISV-PCV connection is disconnected again and the secondary sprout takes part in lymphatic development, whereas strong dorsal flow is established in the arterial ISV.

D) *Tg[tp1-MmHbb:kaede]$^{um15}$* embryo mosaically expressing a *pT2Fli1ep-zN1aICD-basfli-mCherry* construct (NICD$^{OE}$) at 52 hpf. Endogenous Notch activity was blocked by treatment with 25µM DAPT from 24 till 52 hpf in order to observe Notch activation by NICD overexpression.

E) Quantification of the ratio of arterial and venous ISVs containing cells overexpressing Su(H)VP16, a constitutively active variant of the Su(H) transcription factor (Su(H)VP16$^{OE}$) (N=3 experiments, 29 embryos) or mosaically overexpressing Notch-1bICD (N1bICD$^{OE}$), the intracellular domain of Notch1b, the paralogue of Notch1a (N=2 experiments, 43 embryos). (*P=0.0001)

F) Stills from time-lapse movie (Supplementary Movie S3) in *Tg[fli1a:GFP]$^{y1}$* embryos mosaically overexpressing a *pTol2-zN1aICD-basfli-mCherry* construct showing formation of a transient perfused three-way connection between a wild-type secondary sprout and a NICD overexpressing (NICD$^{OE}$) primary ISV, indicating that Notch activation does not prevent interaction between primary ISVs and secondary sprouts.

Scale bars, 10µm

Development • Supplementary information

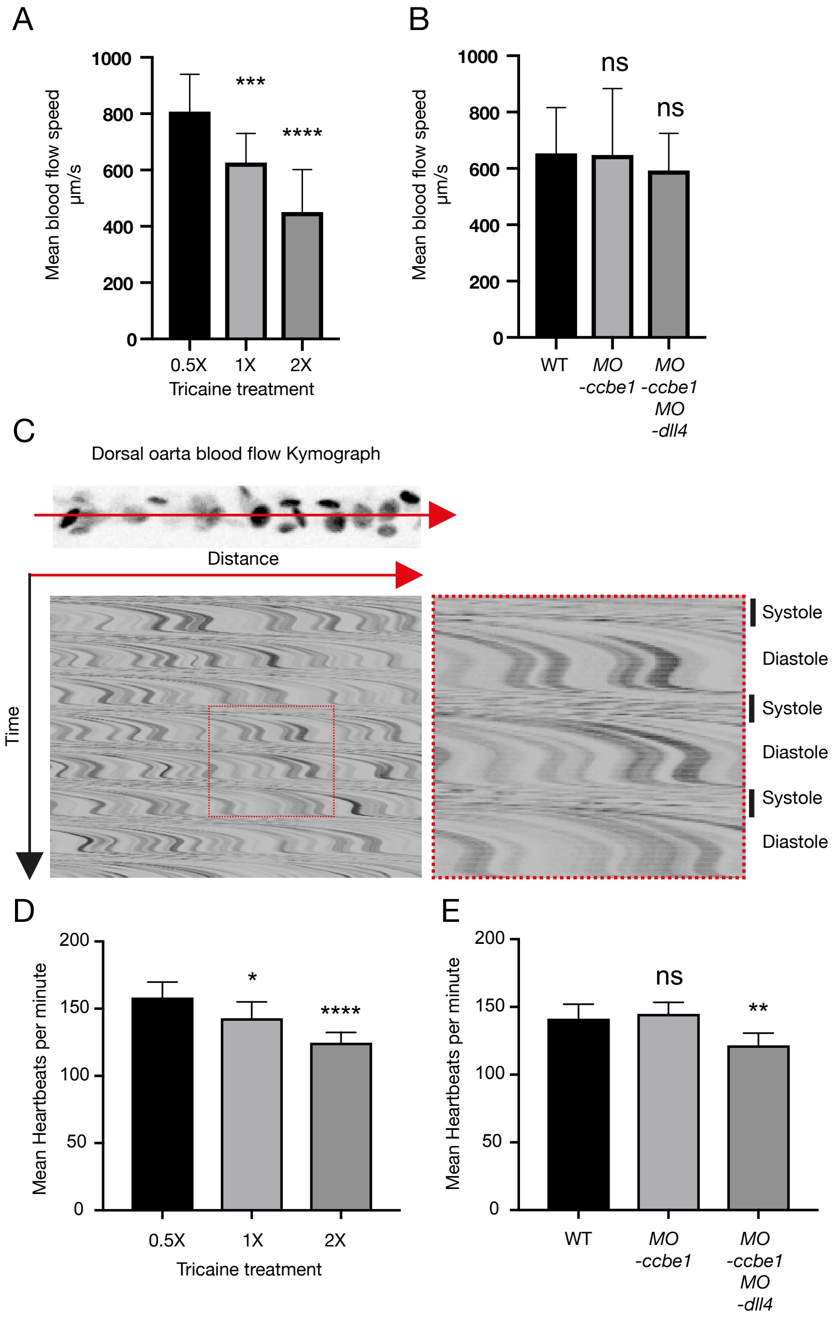


Development • Supplementary information

**Figure S2**

A) Quantification of mean blood flow speed (in μm/s) in the dorsal aorta of *Tg[gata1a:dsRed]*$^{sd2}$ embryos at 50 hpf*,* after 1h incubation at 28.5°C in fish media containing 0.007% (0.5X), 0.014% (1X) or 0.028% (2x) tricaine. (n=16 0.5X tricaine treated embryos, n=18 1X tricaine treated embryos, n=17 2x tricaine treated embryos).

B) Quantification of average blood flow speed (in μm/s) in the dorsal aorta of *Tg[gata1a:dsRed]*$^{sd}$*;Tg[fli1a:GFP]*$^{y1}$ WT, *MO-ccbe1* (5ng) and *MO-dll4* (10ng) embryos at 50 hpf*, in* 0.014% tricaine*.* (n=19 WT, n=22 *MO-ccbe1*, n=7 *MO-dll4*). Only embryos exhibiting no secondary sprouts were used for quantification.

C) Representation of a kymograph displaying red blood cell movement in the dorsal aorta of *Tg[gata1a:dsRed]*$^{sd2}$ embryos.

D) Quantification of heart rate (heartbeats/minute) in the dorsal aorta of *Tg[gata1a:dsRed]*$^{sd2}$ embryos at 50 hpf*,* after 1h incubation at 28.5C in fish media containing 0.007% (0.5X), 0.014% (1X) or 0.028% (2x) tricaine. (n=18 0.5X tricaine treated embryos, n=19 1X tricaine treated embryos, n=19 2x tricaine treated embryos).

E) Quantification of heart rate (heartbeats/minute) in the dorsal aorta of *Tg[gata1a:dsRed]*$^{sd}$*;Tg[fli1a:GFP]*$^{y1}$ WT, *MO-ccbe1* (5ng) and *MO-dll4* (10ng) embryos at 50 hpf*, in* 0.014% tricaine*.* (n=20 WT, n=22 *MO-ccbe1*, n=9 *MO-dll4*). Only embryos exhibiting no secondary sprouts were used for quantification.

Development • Supplementary information

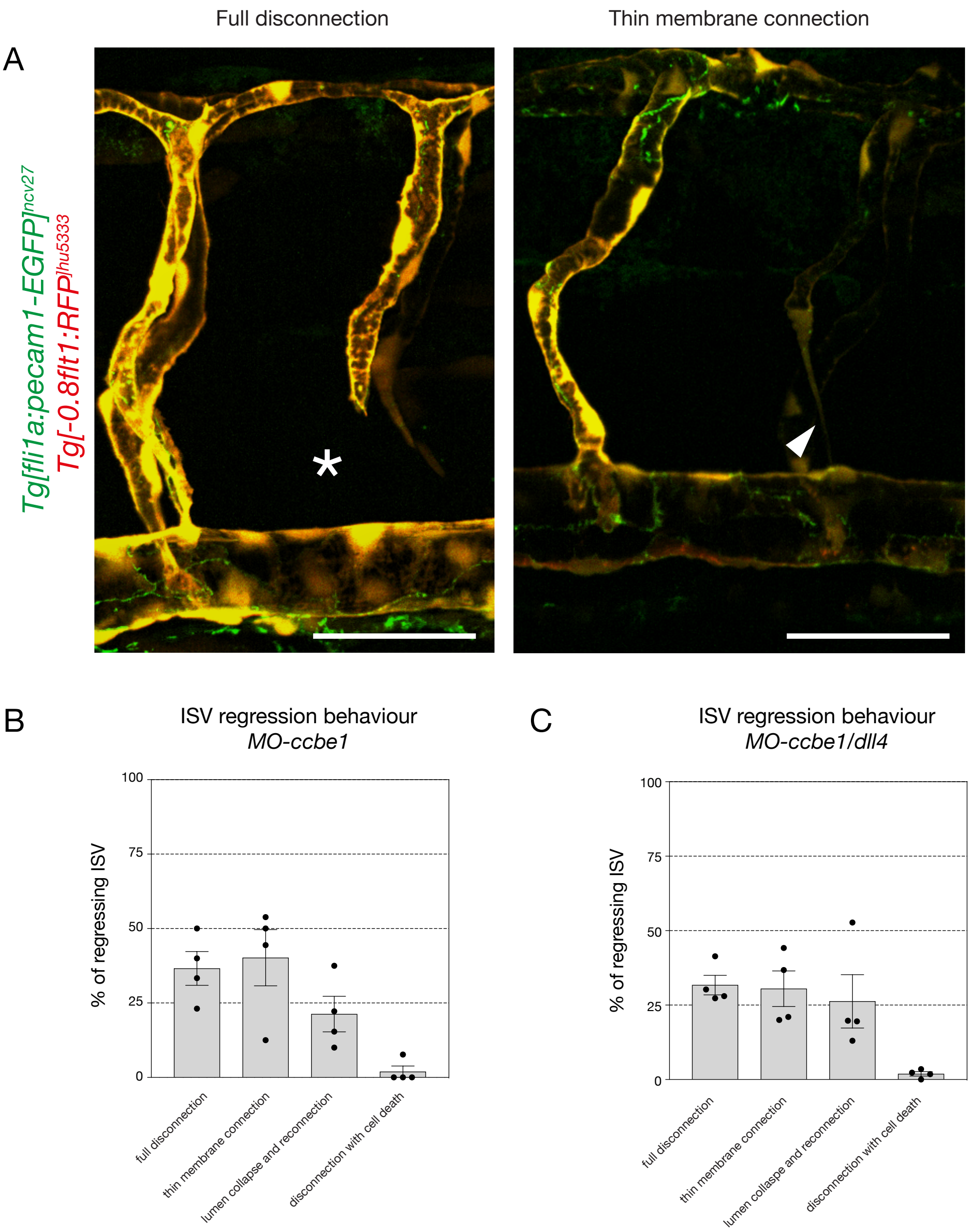


**Figure S3**

A) Example of two different type of regression behaviour, imaged in T*g[fli1a:pecam1-EGFP]*$^{ncv27}$*; Tg[-0.8flt1:RFP]*$^{hu5333}$ 5ng *MO-ccbe1* embryos: Full disconnection and thin membrane connection left.

B) Quantification of the nature of ISV regression behaviour in *MO-ccbe1* embryos (N=4 experiments, 37 embryos).

C) Quantification of the nature of ISV regression behaviour in *MO-ccbe1/MO-dll4* embryos (N=4 experiments, 62 morphants).

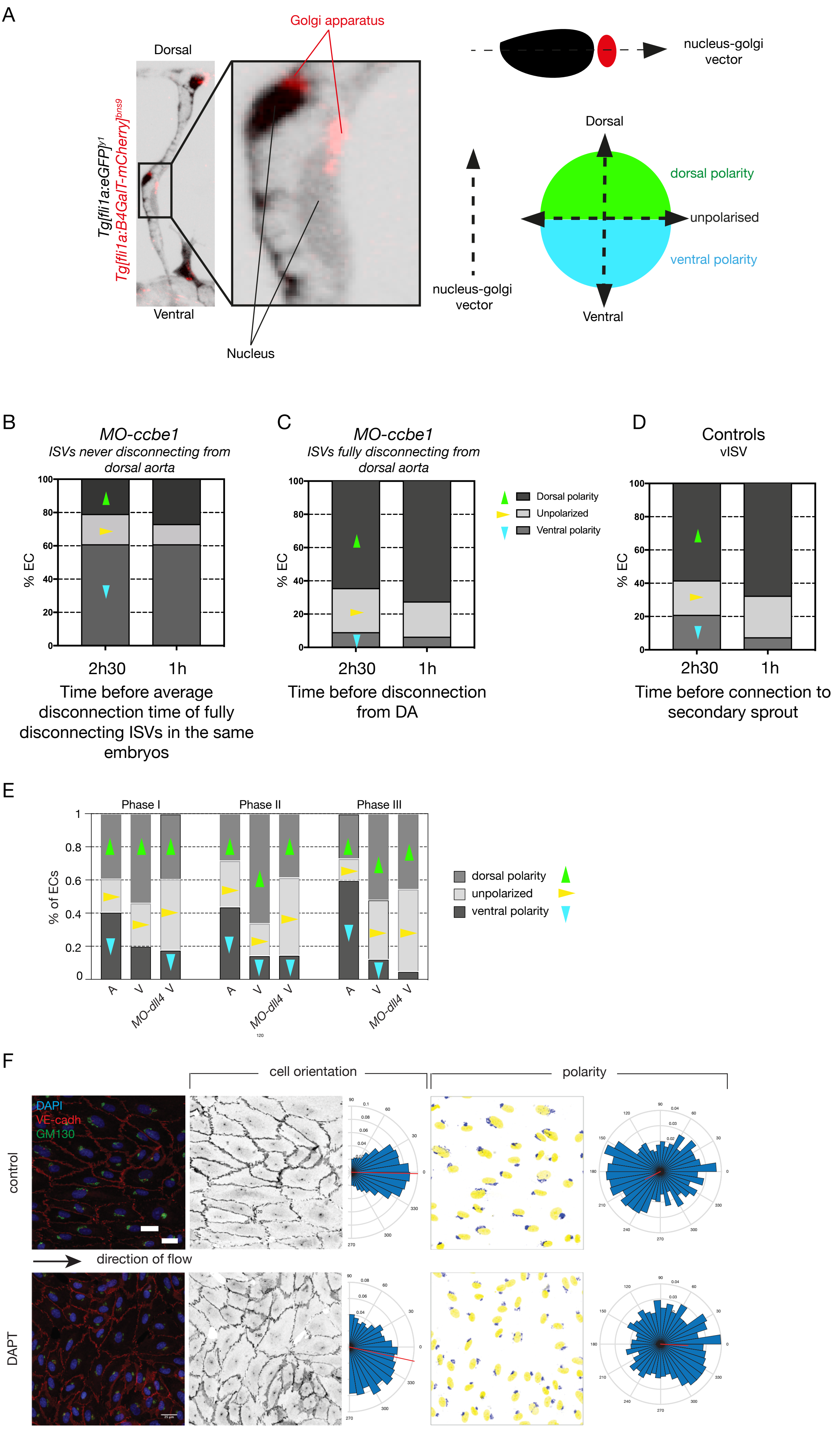
A
Golgi apparatus
Dorsal
Ventral
Nucleus
Tg[fli1a:eGFP]y1
Tg[fli1a:B4GalT-mCherry]bns9
nucleus-golgi vector
Dorsal
dorsal polarity
unpolarised
ventral polarity
Ventral
nucleus-golgi vector
B
MO-ccbe1
ISVs never disconnecting from dorsal aorta
% EC
2h30
1h
Time before average disconnection time of fully disconnecting ISVs in the same embryos
C
MO-ccbe1
ISVs fully disconnecting from dorsal aorta
% EC
2h30
1h
Time before disconnection from DA
Dorsal polarity
Unpolarized
Ventral polarity
D
Controls
vISV
% EC
2h30
1h
Time before connection to secondary sprout
E
Phase I
Phase II
Phase III
% of ECs
A
V
MO-dll4 V
dorsal polarity
unpolarized
ventral polarity
F
cell orientation
polarity
DAPI
VE-cadh
GM130
control
direction of flow
DAPT

**Figure S4**

A) Clarification of the method used to classify to analyse polarity of ECs during vascular remodelling in Figure 3 and Supplementary Figure 4. Time-lapse movies were made of transgenic *Tg[fli1a:GFP]$^{y1}$; Tg[fli1a:B4GalT1-mCherry]$^{bns9}$* embryos during vascular remodelling in the trunk (~32 hpf to ~54 hpf). Polarity arrows from the centre of the nucleus to the centre of the Golgi apparatus were drawn manually. For every primary ISV cells the polarity was scored per time point: dorsal polarity, ventral polarity or unpolarized, depending on the relative position of Golgi and nucleus, i.e. respectively, Golgi dorsal, ventral or parallel to the nucleus in respect to the local angle of the ISV.

B) Quantification of EC polarity in ISV never disconnecting from the dorsal aorta in *Tg[fli1a:Hsa.B4GALT1-mCherry]$^{bns9}$;Tg[fli1a:EGFP]$^{y1}$* embryos injected with 5ng *MO-ccbe1* during presumptive phase I (measured at both 1h and 2h30 prior to the average disconnecting time of ISVs in the same embryos). (n=7 morphants, 12 ISVs, 33 ECs at both 1h and 2h30 before average disconnection time).

C) Quantification of EC polarity in ISVs fully disconnecting from the dorsal aorta in *Tg[fli1a:Hsa.B4GALT1-mCherry]$^{bns9}$;Tg[fli1a:EGFP]$^{y1}$* embryos injected with 5ng *MO-ccbe1* during presumptive Phase I (measured at both 1h and 2h30 before disconnection from the dorsal aorta). (n=7 morphants, 14 ISVs, 33 and 34 ECs at 1h and 2h30 before disconnection from the dorsal aorta, respectively).

D) Quantification of EC polarity in vISVs of *Tg[fli1a:Hsa.B4GALT1-mCherry]$^{bns9}$; Tg[fli1a: EGFP]$^{y1}$* control embryos during phase I. (n=6 fish, 10 ISVs, 28 and 29 ECs at 1h and 2h30 before connection to the secondary sprout, respectively).

E) Quantification of EC polarity in aISVs and vISVs of *Tg[fli1a:EGFP]$^{y1}$;Tg[fli1a:B4GalT-mCherry]$^{bns9}$ MO-dll4* and WT embryos at 3 different time points: I) 2.5 hours before secondary sprout connection, II) during three-way connection and III) 2.5 hours after resolution (n=7 WT aISV, 8 WT vISV, 10 *MO-dll4* vISV).

Development • Supplementary information

F) Flow chamber experiment. A confluent layer of HUVEC cells was exposed to high shear stress and treated with 5µM DAPT or DMSO (control). Cells were stained for DAPI (nuclei), VE-cadherin (cell boundaries) and GM130 (Golgi). Cell orientation was analyzed by plotting the direction of the main axis of the nucleus. Cell polarity was determined by defining the angle between the center of the nucleus and the center of the Golgi. The graphs represent the percentage of cells with a certain angle of direction or polarity relative to the direction of flow (10° intervals), the red line indicates the mean.

Development • Supplementary information

## Table S1

| Figure | test | SD or SEM | n value | number of times experiment was repeated | P value |
|---|---|---|---|---|---|
| Figure 1B | one-way ANOVA + Tukey's multiple comparisons test | SEM | 74 embryos, 1184 ISVs (**A&A: 344; A&V: 526; V&V: 314**) | 3 experiments | ANOVA: P<0.0001 (F=202)<br>A&A vs A&V: P<0.0001<br>A&V vs V&V: P<0.0001 |
| Figure 1C | Unpaired t test with Welch's correction | SEM | 74 embryos, 1480 ISVs (**A: 760; V:720**) | 3 experiments | A vs V: P<0.0001 (2-tailed; t=4.638; df=1475) |
| Figure 1G | one-way ANOVA + Tukey's multiple comparisons test | SEM | WT: **74** embryos<br>NICD OE: **51** embryos | WT: 3 experiments<br>NICD OE: 3 experiments | ANOVA: P<0.0001 (F=493)<br>UIC vs mosaic overall: NS (P=0.9661)<br>mosaic overall vs mosaic NICD OE: P<0.0001<br>mosaic overall vs mosaic WT: P<0.0001 |
| Figure 1H | one-way ANOVA + Tukey's multiple comparisons test | SEM | < 20%: n = **13** embryos<br>20-30%: n = **26** embryos<br>>30%: n = **12** embryos | 3 experiments | ANOVA: P<0.0001 (F=12.91)<br><20% vs 20-30%: NS (P = 0.1672)<br><20% vs >30%: P<0.0001<br>20-30% vs >30%: P=0.0009 |
| Figure 1I | one-way ANOVA + Sidak's multiple comparisons test | SEM | WT: 74 embryos, 1184 ISVs (**A&A: 344; A&V: 526; V&V: 314**)<br>NICD OE: 40 embryos, 592 ISVs (**A&A: 188; A&V: 229; V&V: 174**) | WT: 3 experiments<br>NICD OE: 3 experiments | ANOVA: P<0.0001 (F=74.39)<br>A&A WT vs A&A NICD OE: P<0.001<br>A&V WT vs A&V NICD OE: NS (P=0.5545)<br>V&V WT vs V&V NICD OE: P<0.0001<br>A&A NICD OE vs A&V NICD OE: NS (P>0.9999)<br>A&V NICD OE vs V&V NICD OE: NS (P=0.0510) |
| Suppl Figure 1A | one-way ANOVA + Tukey's multiple comparisons test | SEM | 74 embryos, 888 ISVs (**4A: 27; 3A/1V: 203; 2A/2V: 450; 1A/3V: 193; 4V: 15**) | 3 experiments | ANOVA: P<0.0001 (F=28.2)<br>4xA vs 3xA/1xV: NS (P=0.646)<br>3xA/1xV vs 2xA/2xV: P<0.0001<br>2xA/2xV vs 1xA/3xV: P=0.0009<br>1xA/3xV vs 4xV: NS (P=0.1231) |
| Suppl Figure 1B | Unpaired t test with Welch's correction | SEM | 74 embryos, 1332 ISVs (**V: 642; A: 690**) | 3 experiments | A vs V: P<0.0001 (2-tailed; t=14.91; df=1328) |
| Suppl Figure 1E | one-way ANOVA + Dunnett's multiple comparisons test | SEM | UIC: **74** embryos; Su(H)VP16: **29** embryos, 99 ISVs; N1bICD: **43** embryos, 373 ISVs | 3 experiments | UIC vs Su(H)VP16 OE: P=0.0001<br>UIC vs N1bICD: P=0.0001 |
| Figure 2A | Unpaired t test with Welch's correction | SEM | control: **74** embryos<br>tricaine (2x): **65** embryos | control: 3 experiments<br>tricain: 3 experiments | ctrl vs tricaine: P<0.0001 (2-tailed; t=6.001; df=119.4) |
| Figure 2B | one-way ANOVA + Sidak's multiple comparisons test | SEM | control: 74 embryos, 1184 ISVs (**A&A: 344; A&V: 526; V&V: 314**)<br>tricaine 2x: 65 embryos, 950 ISVs (**A&A: 359; A&V: 407; V&V: 174**) | control: 3 experiments<br>tricain: 3 experiments | ANOVA: P<0.0001 (F=115.5)<br>A&A ctrl vs A&A tric: P<0.001<br>A&V ctrl vs A&V tric: P=0.0002<br>V&V ctrl vs V&V tric: NS (P>0.9999)<br>A&A tric vs A&V tric: P<0.001<br>A&V tric vs V&V tric: P<0.001 |
| Figure 2C | Unpaired t test with Welch's correction | SEM | NICD OE untreated (-): **51** embryos<br>NICD OE + tricain (+): **20** embryos | NICD OE untreated: 3 experiments<br>NICD OE + tricain: 2 experiments | NICD OE (-) vs NICD OE (+): NS (P=0.8859) (2-tailed; t=0.1826; df=48.15)<br>WT (-) vs WT (+): P<0.0001 (2-tailed; t=5.364; df=39.74)<br>overall (-) vs overall (+): P=0.0003 (2-tailed; t=3.955; df=37.16) |
| Figure 3E | Chi-square | N/A | aISV: 7 ISVs, 16 cells<br>vISV: 8 ISVs, 17 cells | 6 experiments | phase I A vs V: P=0.0075 (X2=9.782; df=2)<br>phase II A vs V: P<0.0001 (X2=152.7; df=2)<br>phase III A vs V: P<0.0001 (X2=74.22; df=2) |
| Figure 3F | Unpaired t test with Welch's correction | SEM | aISV: 12 ISVs, 67 cells (**I: 48; II: 54; III: 67**)<br>vISV: 13 ISVs, 103 cells (**I: 49; II: 54; III: 93**) | 5 experiments | phase I A vs V: P=0.0032 (t=2.177; df=94.94)<br>phase II A vs V: P=0.0169 (=2.429; df=101.5)<br>phase III A vs V: P=0.0213 (t=2.328; df=147.4) |
| Figure 3H | Chi-square | N/A | 34 embryos, **12** aISVs, **40** vISVs | 5 experiments | A vs V: P<0.0001 (X2=23.92; df=2) |
| Figure 3J | N/A | SEM | **37** morphants, **241** morphant vessels | 4 experiments | N/A |
| Figure 3K | N/A | SEM | **37** morphants, **29** WT controls | 4 experiments | N/A |
| Figure 3M | N/A | SEM | **62** morphants, **531** morphant vessels | 7 experiments | N/A |
| Figure 3N | N/A | SEM | **62** morphants, **17** WT controls | 4 experiments | N/A |
| Figure 4C | N/A | N/A | **13** embryos, 23 vISV, 11 aISVS | 4 experiments | N/A |
| Suppl Figure 2A | Brown-Forsythe and Welch Anova test ANOVA | SD | **16** embryos (0.5X treatment), **18** embryos (1X treatment), **17** embryos (2x treatment) | 2 experiments | 0.5X vs 1X (P=0.0003), 0.5X vs 2X (P<0.0001) |
| Suppl Figure 2B | Brown-Forsythe and Welch Anova test ANOVA | SD | **19** WT controls, **22** ccbe1 morphants, **7** dll4/ccbe1 morphants | 2 experiments | WT vs MO-ccbe1 (P=0.9949), WT vs MO-ccbe1/MO-dll4 (P=0.5498) |

| | | | | | |
|---|---|---|---|---|---|
| Suppl Figure 2D | one way ANOVA with Kruskall-Wallis test | SD | **18** embryos (0.5X treatment), **19** embryos (1X treatment), **19** embryos (2x treatment) | 2 experiments | 0.5X vs 1X (P=0.0129), 0.5X vs 2X (P<0.0001) |
| Suppl Figure 2E | one way Anova with Kruskal Wallis test | SD | **20** WT controls, **22** ccbe1 morphants, **9** dll4/ccbe1 morphants | 2 experiments | WT vs MO-ccbe1 (P=0.9826), WT vs MO-ccbe1/MO-dll4 (P=0.0010) |
| Suppl Figure 3B | N/A | SEM | **37** morphants, **241** morphant vessels | 4 experiments | N/A |
| Suppl Figure 3C | N/A | SEM | **62** morphants, **531** morphant vessels | 7 experiments | N/A |
| Suppl Figure 4B | N/A | N/A | 7 ccbe1 morphants, 12 ISVs, 33 ECs (2h30), 33 Ecs (1h) | 3 experiments | N/A |
| Suppl Figure 4C | N/A | N/A | 7 ccbe1 morphants, 14 ISVs, 34 ECs (2h30), 33 ECs (1h) | 3 experiments | N/A |
| Suppl Figure 4D | N/A | N/A | 6 WT controls, 12 ISVs, 29 ECs (2h30), 28 ECs (1h) | 3 experiments | N/A |
| Suppl Figure 4E | Chi-square | N/A | 7 WT aISV (16 cells), 8 WT vISV (17 cells), 10 Dll4 KD vISV (31 cells) | Dll4 KD: 2 experiments | V I vs Dll4 KD I: P=0.0006 (X2=14.95; df=2)<br>V II vs Dll4 KD II: P<0.001 (X2=116.5; df=2)<br>V III vs Dll4 KD III: P=0.0010 (X2=13.77; df=2) |
| Suppl Figure 4F | Kuiper two-sample test | N/A | **5** experiments (min 1500 cells/exp) | 5 experiments | cell orientation: ctrl vs DAPT: P=0.0001<br>cell polarity: ctrl vs DAPT : P=0.0001 |
| Figure 5A | one-way ANOVA + Tukey's multiple comparisons test | SEM | WT: 12 aISV (**I: 48; II: 54; III: 67**), 13 vISV (**I: 49; II: 54; III: 93**)<br>NICD OE: 30 aISV, 29 cells (**I: 29; II: 9; III: 13**)<br>Dll4 KD: 9 vISV, 85 cells (**I: 53; II: 85;III: 45**) | WT: 5 experiments<br>NICD OE: 4 experiments<br>Dll4 KD: 2 experiments | ANOVA phase I: P=0.0071 (F=4.154)<br>ANOVA phase II: P=0.0001 (F=7.221)<br>ANOVA phase III: P=0.0003 (F=6.623)<br>A I vs NICD OE I: NS (P=0.9923)<br>A II vs NICD OE II: NS (P=0.8909)<br>A III vs NICD OE III: NS (P=0.9447)<br>V I vs Dll4 KD I: NS (P>0.9999)<br>V II vs Dll4 KD II: NS (P=0.0956)<br>V III vs Dll4 KD III: NS (P>0.9999) |
| Figure 5D | 2way ANOVA + Tukey's multiple comparisons test | SEM | **20** embryos | 5 experiments | tp1 neg vs tp1pos: P<0.0001 |
| Figure 5E | Paired t-test | SEM | control: **20** embryos; tricaine 2x: **12** embryos | control: 5 experiments<br>tricaine 2x: 3 experiments | control vs tric 2x: NS (P>0.9999) |
| Figure 5F | 2way ANOVA + Tukey's multiple comparisons test | SEM | control: **20** embryos; tricaine 2x: **12** embryos | control: 5 experiments<br>tricaine 2x: 3 experiments | tp1 pos ctrl vs tp1 pos tric2x: NS (P=0.9923)<br>tp1 neg ctrl vs tp1 neg tric2x: P=0.0269 |
| Figure 5G | one-way ANOVA + Sidak's multiple comparisons test | SEM | WT: 19 embryos, 199 ISVs (**0&0: 78; 0&1: 83; 1&1: 38**) | 2 experiments | ANOVA: P<0.0001 (F=12.19)<br>0&0 vs 0&1: P=0.0035<br>0&0 vs 1&1: P<0.0001<br>0&1 vs 1&1: NS (P=0.1065) |

Development • Supplementary information

## Supplementary Movies

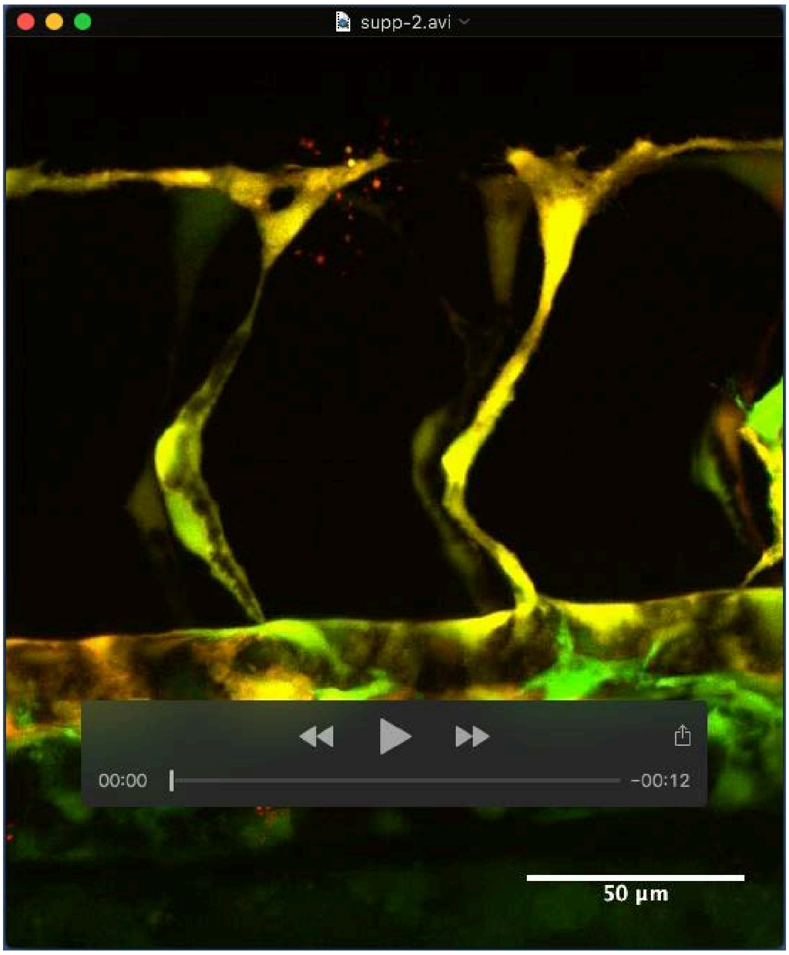


**Movie** 1 Time-lapse imaging of a *Tg[fli1a:EGFP]*$^{y1}$;*Tg[-0.8flt1:RFP]*$^{hu5333}$ embryo (all ECs labeled in green, arterial ECs labeled in red) showing ISV remodelling into a venous (left) and an arterial (right) intersegmental vessel from 26 to 55 hpf (frame interval: 15 minutes). In both cases, a lumenized connection is formed between the secondary sprout and the primary ISV. In the case of the formation of an aISV, the connection is lost again and the secondary sprouts forms lymphatic precursors at the horizontal myoseptum (Parachordal lymphangioblasts). In case of vISV remodelling, the secondary sprout connection is stabilized and the connection between primary ISV and DA regresses. Scale bars, 50µm

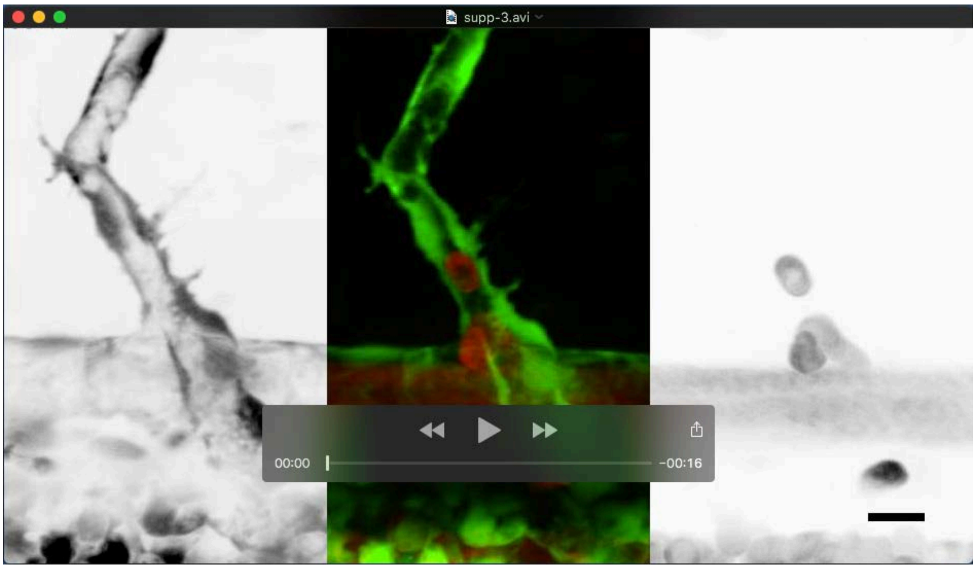


Movie 2 Time-lapse recording of a *Tg[fli1a:EGFP]*$^{y1}$;*Tg[gata1a:DsRed]*$^{sd2}$ embryo (with ECs labeled in green and erythrocytes in red) between 35 and 40 hpf (frame interval: 2 minutes) showing a transient perfused three-way connection in which the ISV-PCV connection dissociates again to form an arterial ISV and a lymphangiogenic sprout. Scale bars, 10µm

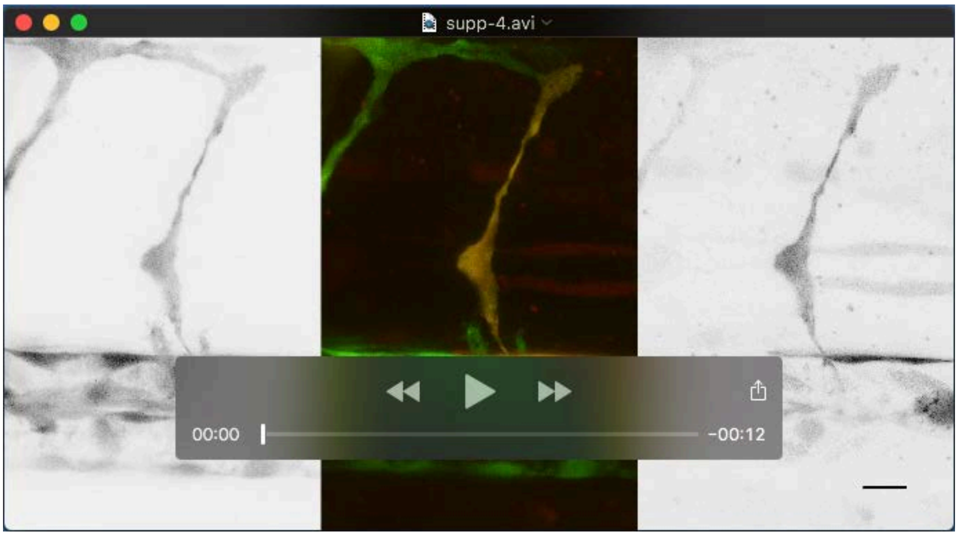


Movie 3 Time-lapse recording of a *Tg[fli1a:EGFP]*$^{y1}$ embryo (labeling ECs in green) mosaically overexpressing a pT2-zN1aICD-basfli-mCherry construct (orange-red) between 31 and 43hpf (frame interval: 6 minutes) showing formation of a transient perfused three-way connection between a wild-type secondary sprout and a NICD$^{OE}$ primary ISV. Scale bar, 15µm

Development • Supplementary information

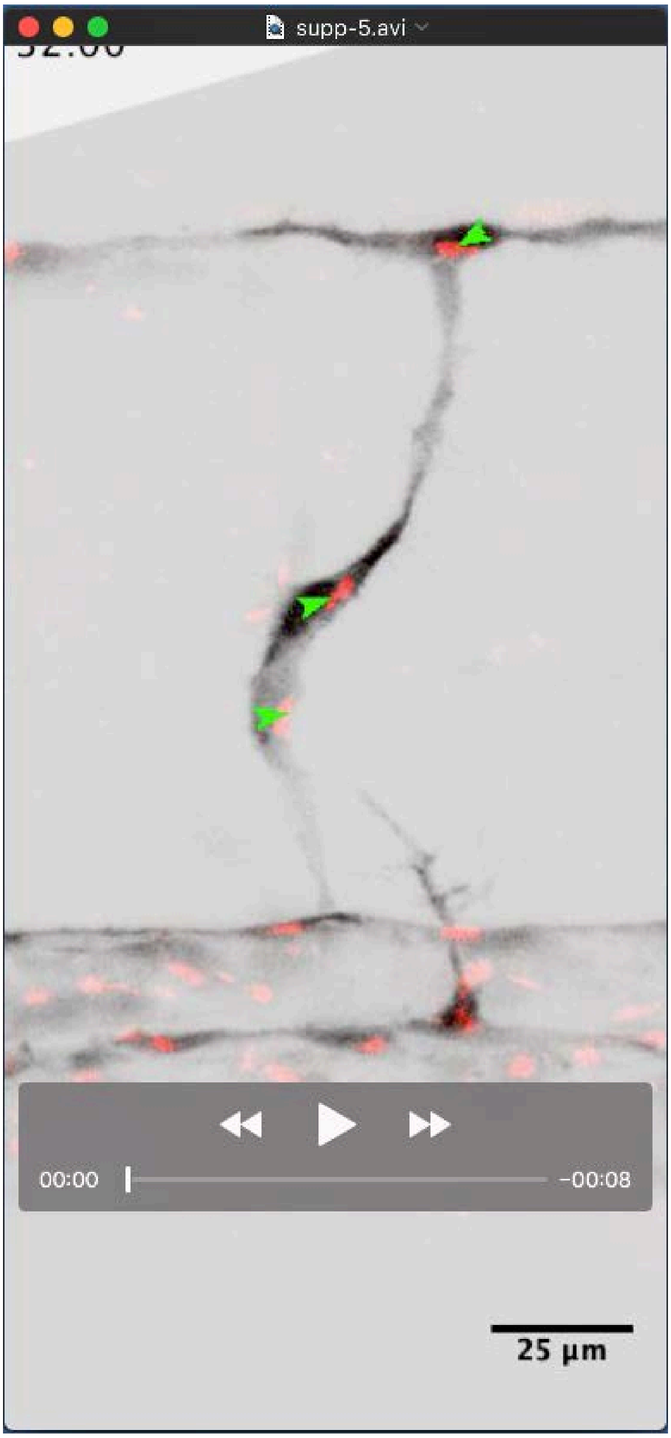


Movie 4 Time-lapse imaging of a *Tg[fli1a:EGFP*$^{y1}$*;Tg[fli1a:B4GalT-mCherry]*$^{bns9}$ embryo (ECs depicted in grey, endothelial Golgi apparatus in red) showing EC polarity in an arterial ISV from 32 to 51 hpf. Arrows (green in primary ISV, blue in secondary sprout) point from the centre of the nucleus to the centre of the Golgi complex (frame interval: 15 minutes). Scale bars, 25µm

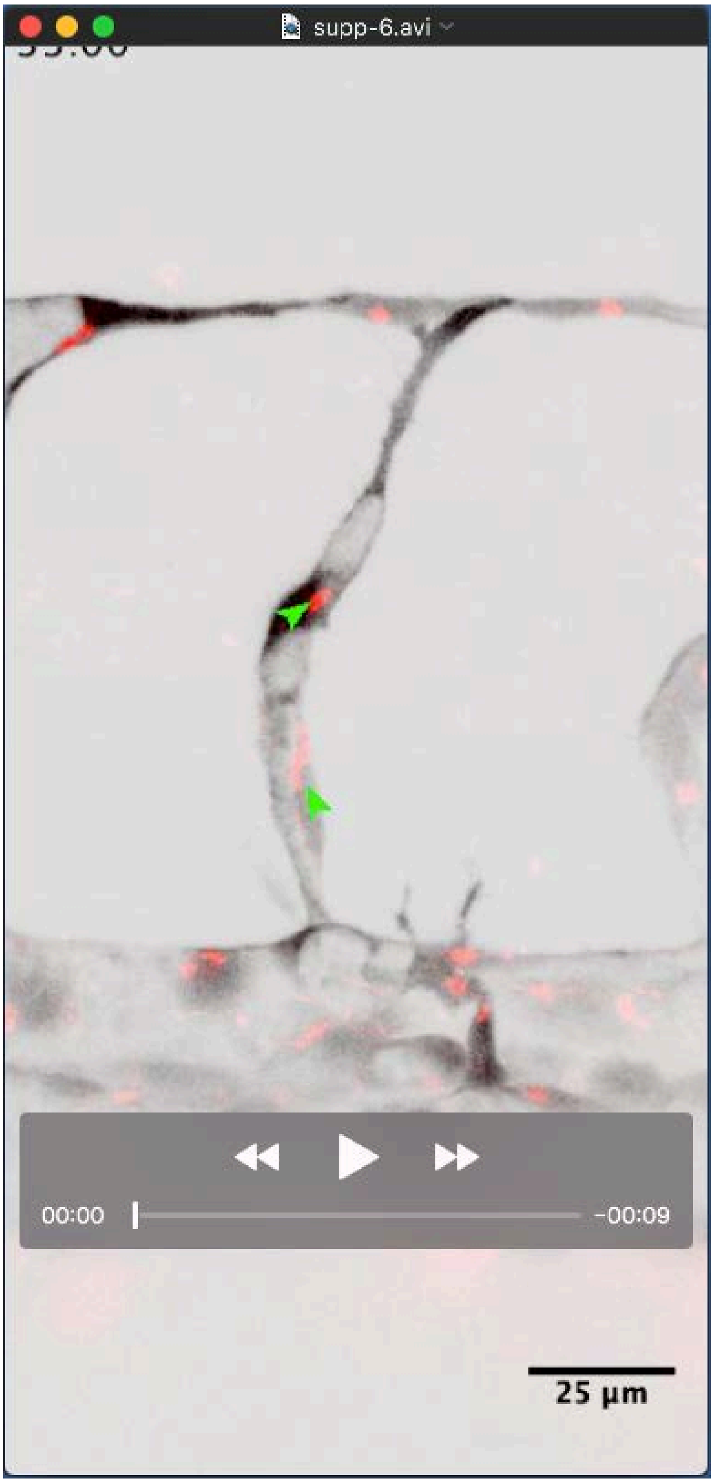


Movie 5 Time-lapse imaging of *a Tg[fli1a:EGFP]$^{y1}$;Tg[fli1a:B4GalT-mCherry]$^{bns9}$* embryo (ECs depicted in grey, endothelial Golgi apparatus in red) showing EC polarity in a venous ISV from 33 to 55 hpf. Arrows (green in primary ISV, blue in secondary sprout) point fromthe centre of the nucleus to the centre of the Golgi complex (frame interval: 15 minutes). Scale bars, 25µm

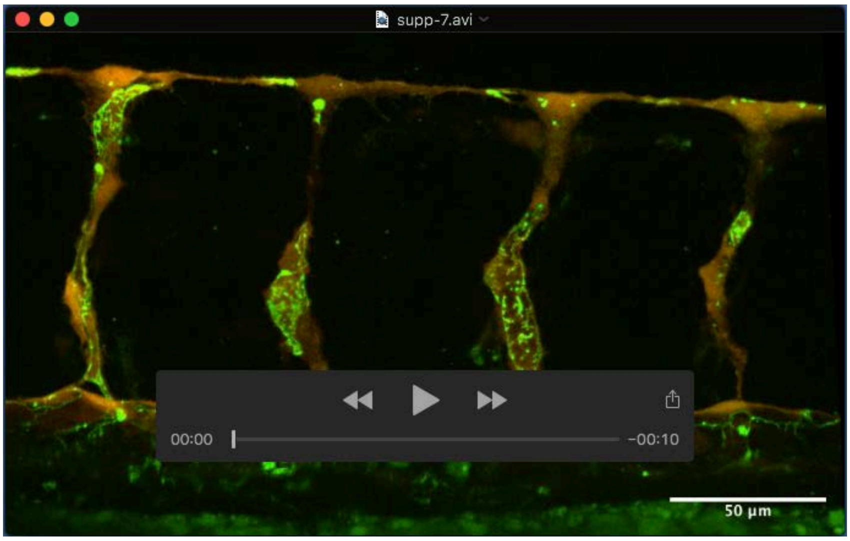


Movie 6 Time-lapse imaging of *Tg[fli1a:pecam1-EGFP]$^{ncv27}$;Tg[-0.8flt1:RFP]$^{hu5333}$* embryo (junctions in green, arterial structures in red) from 29 to 53 hpf showing ISV remodelling into arterial and intersegmental vessels (aISV and vISV)(frame interval: 15 minutes). Scale bars, 50µm

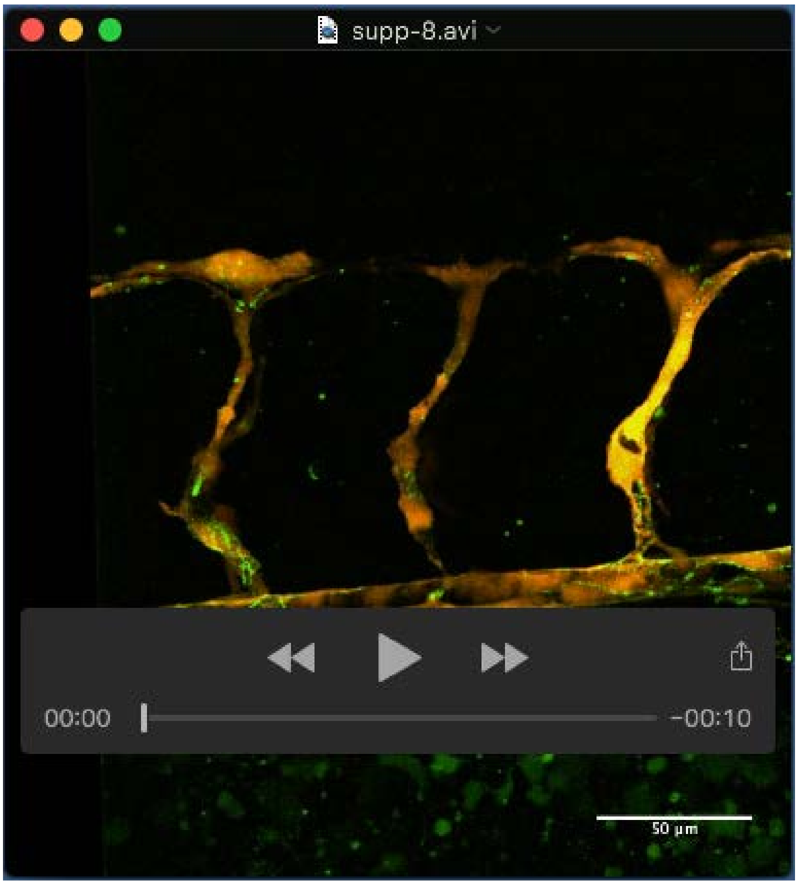


Movie 7 Time-lapse imaging of a *Tg[fli1a:pecam1-EGFP]$^{ncv27}$;Tg[-0.8flt1:RFP]$^{hu5333}$ MO-ccbe1* embryo (junctions in green, arterial structures in red) from 30 to 54h30 hpf showing ISV regression in the absence of secondary sprouting (frame interval: 15 minutes). Scale bars, 50µm

Development • Supplementary information

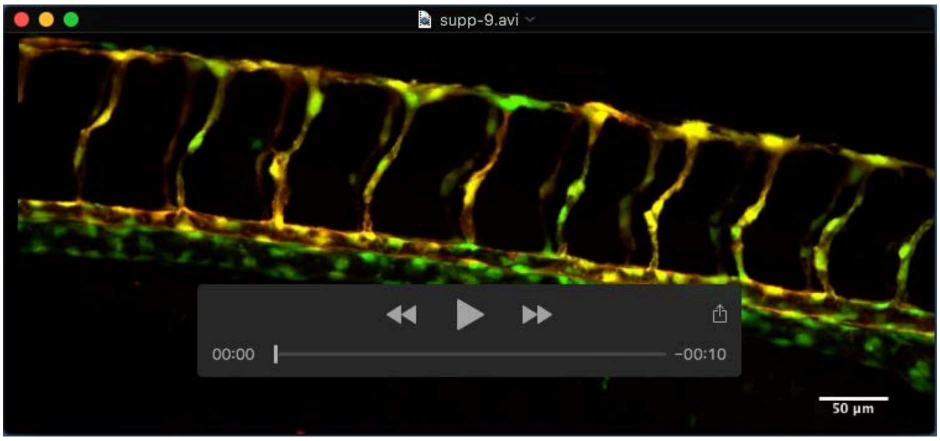


Movie 8 Time-lapse imaging of a *Tg[fli1a:EGFP]*$^{y1}$*;Tg[-0.8flt1:RFP]*$^{hu5333}$ *MO-ccbe1/MO-dll4* embryo (all ECs labeled in green, arterial ECs labeled in red) from 30 to 54h30 hpf showing ISV regression in the absence of secondary sprouting (frame interval: 15 minutes). Scale bars, 50µm

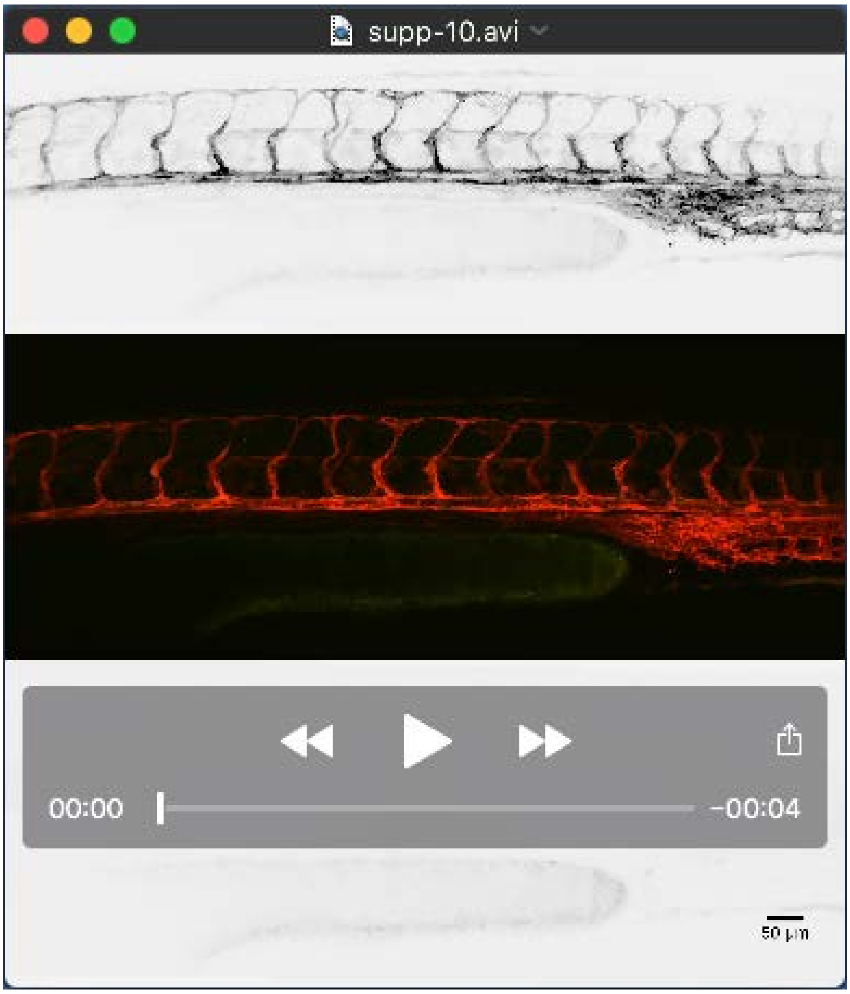


Movie 9 Time-lapse imaging of the Notch activity reporter *Tg[tp1-MmHbb:kaede]*$^{um15}$;*Tg[kdr-l:ras-Cherry]*$^{s916}$ imaged from 30 hpf (right after conversion of the Kaede$^{green}$ protein into Kaede$^{red}$) to 50hpf. The Notch activity reporter is shown in kaede (red: old signal after conversion/green: new signal), all ECs are labeled in red) (frame interval: 45 minutes). Scale bars, 50µm

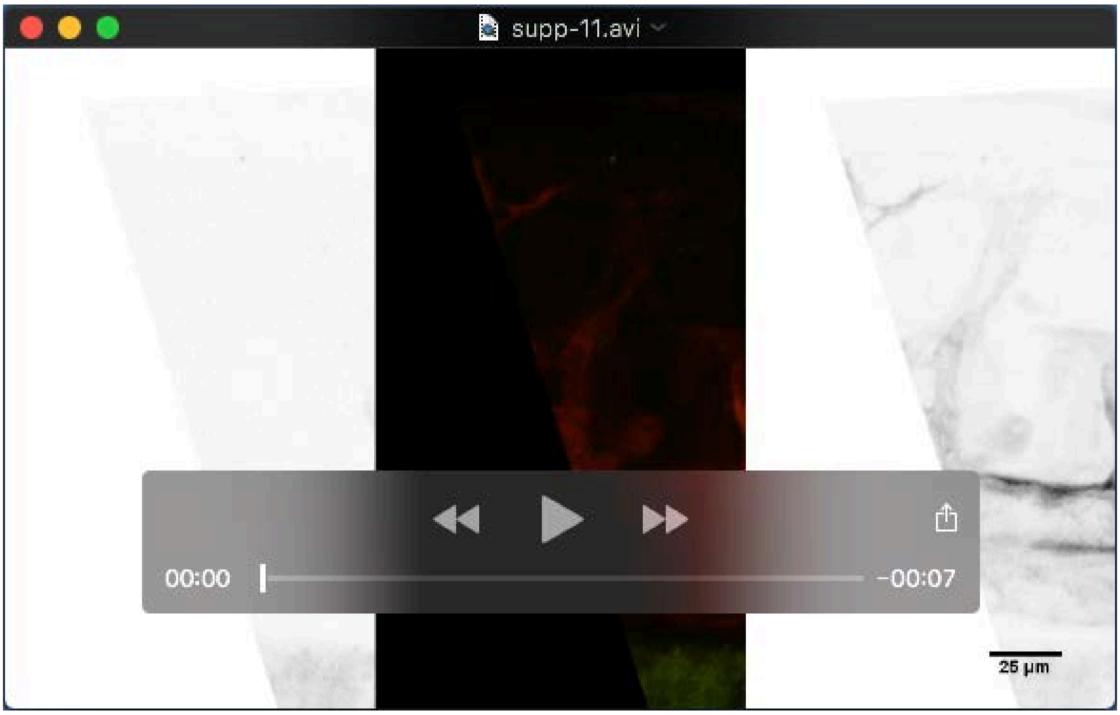


Movie 10 Time-lapse imaging of the Notch activity reporter *Tg[tp1-MmHbb:kaede]$^{um15}$;Tg[kdr-l:ras-Cherry]$^{s916}$* in a future arterial ISV, imaged from 29 hpf (right after conversion of the Kaede$^{green}$ protein into Kaede$^{red}$) to 48,5 hpf. The Notch activity reporter is shown in kaede (red: old signal after conversion/green: new signal), all ECs are labeled in red). The tip of the growing secondary sprout is indicated by arrows. The tp1 signal (Notch activity) builds up before connection of the secondary sprout. (frame interval: 30 minutes). Scale bars, 25µm
